# Classes of non-conventional tetraspanins defined by alternative splicing

**DOI:** 10.1038/s41598-019-50267-0

**Published:** 2019-10-01

**Authors:** Nikolas Hochheimer, Ricarda Sies, Anna C. Aschenbrenner, Dirk Schneider, Thorsten Lang

**Affiliations:** 1 Department of Membrane Biochemistry, Life & Medical Sciences Institute (LIMES), University of Bonn, Carl-Troll-Straße 31, 53115 Bonn, Germany; 2 Department of Genomics and Immunoregulation, Life & Medical Sciences Institute (LIMES), University of Bonn, Carl-Troll-Straße 31, 53115 Bonn, Germany; 3 Department of Internal Medicine and Radboud Center for Infectious Diseases (RCI), Radboud University Nijmegen Medical Center, Geert Grooteplein Zuid 8, Nijmegen, 6525 GA The Netherlands; 4 Institute of Pharmacy and Biochemistry, Johannes Gutenberg University Mainz, Johann-Joachim-Becher-Weg 30, 55128 Mainz, Germany

**Keywords:** RNA splicing, Proteomics, Structural biology

## Abstract

Tetraspanins emerge as a family of membrane proteins mediating an exceptional broad diversity of functions. The naming refers to their four transmembrane segments, which define the tetraspanins‘ typical membrane topology. In this study, we analyzed alternative splicing of tetraspanins. Besides isoforms with four transmembrane segments, most mRNA sequences are coding for isoforms with one, two or three transmembrane segments, representing structurally mono-, di- and trispanins. Moreover, alternative splicing may alter transmembrane topology, delete parts of the large extracellular loop, or generate alternative N- or C-termini. As a result, we define structure-based classes of non-conventional tetraspanins. The increase in gene products by alternative splicing is associated with an unexpected high structural variability of tetraspanins. We speculate that non-conventional tetraspanins have roles in regulating ER exit and modulating tetraspanin-enriched microdomain function.

## Introduction

Tetraspanins are small membrane proteins expressed in all multicellular eukaryotes. With a few exceptions, they are localized at the plasma membrane^1^. Tetraspanins are plasma membrane (PM) master organizers or scaffolding proteins^2,3^. They interact with one another and integrins, immunoglobulin superfamily proteins, proteases and receptors. By these interactions they laterally associate a set of components into so-called tetraspanin-enriched microdomains (TEMs)^4^. Additionally, tetraspanin interactions in the ER are required for co-transport of proteins from the endoplasmatic reticulum (ER) to the PM, a mechanism depending on TEMs already assembling in the ER^5^. Depending on the cell type and the group of associated proteins, TEMs mediate many different functions^6^. As a result, tetraspanins regulate trafficking, signaling, cell proliferation, adhesion, spreading, migration, cell-cell fusion, pathogen entry, cancer and other diseases^4,7^.

The human genome encodes 33 tetraspanins (Tspans) systematically named Tspan1 – Tspan33^8^. However, the systematic nomenclature is rarely applied to frequently studied tetraspanins as CD9 (Tspan29), CD63 (Tspan30), CD81 (Tspan28), CD82 (Tspan27), and CD151 (Tspan24). These historic names refer to their identification by the “cluster of differentiation (CD)” protocol. Other historic names are for example uroplakin 1A and 1B (Tspan21 and Tspan20), peripherin-2 (Tspan22) or rod outer segment membrane protein (Tspan23) (for a complete list of non-systematic names see Table S1).

**Figure 1:**
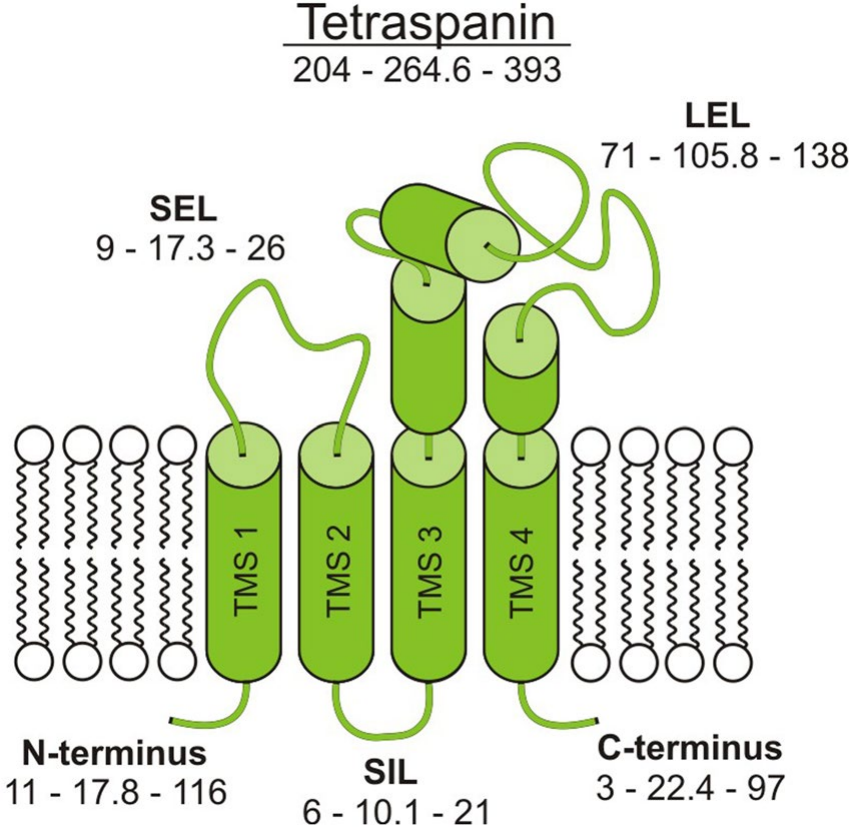
Conventional tetraspanin topology. Depicted is the typical topology of a tetraspanin. Intracellular domains include the N-terminus, the small intracellular loop (SIL), and the C-terminus, which are all short (for exceptions see Table S1). At the extracellular site, a small extracellular loop (SEL) connects transmembrane segment 1 (TMS1) and TMS2 and a large extracellular loop (LEL) TMS3 and TMS4. For the complete tetraspanin and its different segments, the three numbers (xx-yy-zz) indicate the sequence lengths of the shortest sequence (xx), the average sequence (yy) and the longest sequence (zz) (for details see Table S1).

Structurally, all tetraspanins share the same topology (Fig. 1). Thus, in a typical tetraspanin about a third of the protein sequence (see also Table S1) orders into the four transmembrane segments (TMSs), which form two transmembrane helical hairpins. On the extracellular site, a small extracellular loop (SEL) connects the first and the second TMS, and a large extracellular loop (LEL) the third and the fourth TMS^1^. For the SEL, no structural data is available, even in the crystal structure of a full-length Tetraspanin^9^. In contrast, LEL crystal structures^9,10^ reveal five largely α-helical segments (α - ε)^11^ forming a compact structure. The LEL subdivides into a conserved (α, β and ε) and a variable domain (γ and δ). The differences in the variable domains explain the specificity of interactions between tetraspanins and their primary binding partners^12,13^. However, interactions are not restricted to the LEL, but also involve the transmembrane segments and the C-terminus^14^. The N- and C-terminus and the TMS2-TMS3-linker, called small intracellular loop (SIL), are located at the intracellular site. They are short segments containing putative sorting motifs^5^ and palmitoylation sites which stabilize tetraspanin protein interactions^15^. Only five tetraspanins have large C-terminal domains (by definition that their C-termini are two-fold larger than the average; Table S1), from which one has in addition a large N-terminal domain. To date, little is known about their role. For instance, in PRPH-2 an amphipathic helix within the C-terminus partitions into the cytosolic membrane leaflet mediating curvature^16^.

As typical in eukaryotes, *tspan* genes have alternating sections of exons (coding) and introns (non-coding)^17^. From a precursor mRNA, the introns are “spliced out”, yielding the mature mRNA for translation into the protein^18^. Splicing is catalyzed by the spliceosome either co-transcriptionally during transcription, or immediately afterwards. Moreover, self-splicing introns do exist^19^. Yet, the splice product is not necessarily well defined and a pre-mRNA may undergo several splicing pathways, called alternative splicing (AS)^20^. In fact more than 90% of all human genes are subject to AS^21^. Therefore, AS increases the number of gene products^22^. In human, AS leads to more than 80,000 transcripts encoded in the 20,000 human genes^23^. This equals a 4-fold increase in transcriptome diversity, with possible effects in the regulation of protein function.

In contrast to soluble proteins, only little is known about AS of mRNAs coding for membrane integral proteins^24^. Yet, at least ¼ of all open reading frames in any organism code for membrane proteins^25,26^. Compared to soluble proteins, AS could have a stronger impact on the function of membrane proteins. For instance, in eukaryotes, most membrane proteins integrate co-translationally into the membrane at the Sec translocon^27^. Here, the nascent polypeptide chain enters or crosses the membrane. Statistics show that cytosolic segments near the TMS are more positively charged when compared to extracellular segments (positive-inside rule; negative inside depletion/outside enrichment rule^28,29^). This opens the possibility that AS changes the charge distribution and consequently membrane topology. Moreover, certain features of membrane proteins are required for subsequent trafficking from the ER via the Golgi network to the plasma membrane^30^. In the case of tetraspanins, ER retention is caused by truncation of a TMS in CD82^31^, malfunctions in post translational modifications of Tspan1^32^, or by Tspan5 mutants folding improperly^33^. However, proper folding may not be sufficient for ER exit, because deletion of the first CD82 TMS precludes ER exit although the LEL has folded properly. Transport is only restored when the TMS is co-expressed as a separate peptide^31^. Altogether, it is likely that AS eliminates sections required for efficient ER exit.

In the following, we have analyzed the variety of AS of tetraspanin pre-mRNA from the human genome that may enrich the tetraspanin gene products.

## Results and Discussion

We screened the National Center for Biotechnology Information (NCBI) data bank for human tetraspanin gene products. Taking into account only validated and reviewed sequences, we identified 86 mRNAs originating from the 33 human tetraspanin genes. In addition, we found via PCR the sequence of two novel mRNAs, one from a human whole brain and one from a natural killer cells cDNA library (Fig. S1).

Finally, we included a splice variant of CD82 described in the literature^34^. In total, the 89 gene products include the known 33 conventional tetraspanin proteins and 31 different, non-conventional isoforms. The non-conventional isoforms originate from 18 conventional tetraspanins. For Tspan17, we found the highest number of five isoforms (Table 1).

**Table 1:** Tetraspanin splice variants.

Historic Name	Systematic Name	mRNA (NM_)	Protein (NP_)	Isoform	5′UTR	ORF Exons	3′UTR	uORF	Alternative stop codon	Frame shift
	Tspan1	005727.4	005718.2	1	1–3	3–9	9	X		
	Tspan2	005725.6	005716.2	1	1	1–8	8			
		001308315.1	001295244.1	2		Δ4				
		001308316.1	001295245.1	3		Δ7				
	Tspan3	005724.6	005715.1	1	1	1–7	7			
		198902.3	944492.1	2		Δ3				
		001168412.2	001161884.1	3		Δ2				
	Tspan4	001025237.1	001020408.1	1	1–3	3–9	9	X		
		001025234.1	001020405.1	1	↔1					
		001025235.1	001020406.1	1	↔1					
		001025236.1	001020407.1	1	↔1					
		003271.5	003262.1	1	↔1					
		001025238.2	001020409.1	1	↔1, Δ2					
		001025239.1	001020410.1	2	Δ1, Δ3	Δ3				
	Tspan5	005723.4	005714.2	1	1	1–8	8			
	Tspan6	003270.4	003261.1	1	1	1–7	7–8			
		001278740.2	001265669.1	2	1^ASS^	1^ASS^		X		
		001278741.1	001265670.1	2	1^ASS^	1^ASS^		X		
		001278742.1	001265671.1	3	1^ASS^	1^ASS^, Δ7	Δ7	X	Δ7	
		001278743.1	001265672.1	4	1^ASS^	1^ASS^, Δ6		X		
	Tspan7	004615.3	004606.2	1	1	1–7	7–8			
	Tspan8	004616.3	004607.1	1	1–2	2–9	9			
		001369760.1	001356689.1	1	Δ1, 2^ASS^					
	Tspan9	006675.5	006666.1	1	1–3	3–9	9			
		001168320.1	001161792.1	1	Δ2					
	Tspan10	001290212.1	001277141.1	1	1	1–4	4			
		031945.4	114151.3	2	Δ1	Δ1				
	Tspan11	001080509.2	001073978.1	1	1–2	2–8	8	X		
		001370301.1	001357230.1	2	Δ2	Δ2				
		001370302.1	001357231.1	1	↔1					
	Tspan12	012338.4	036470.1	1	1–2	2–8	8			
	Tspan13	014399.4	055214.1	1	1	1–6	6			
	Tspan14	030927.3	112189.2	1	1–2	2–9	9			
		001128309.2	001121781.1	2		Δ3–5				
		001351266.1	001338195.1	1	+1					
		001351267.3	001338196.1	1	+1					
		001351268.1	001338197.1	1	+1					
		001351269.1	001338198.1	1	↔1					
		001351270.1	001338199.1	1	↔1			X		
		001351271.1	001338200.1	1	↔1					
		001351272.1	001338201.1	1	↔1					
	Tspan15	012339.5	036471.1	1	1	1–8	8			
		001351263.1	001338192.1	2		Δ2–3				
	Tspan16	001282509.2	001269438.1	1	1	1–7	7			
		012466.4	036598.1	2		↔7			↔7	↔7
		001282510.2	001269439.1	3		Δ3				
	Tspan17	012171.3	036303.1	1	1	1–9	9			
		130465.5	569732.2	2		6^ASS^				
		001006616.3	001006617.2	3		6^ASS^, 8^ASS^			8^ASS^	8^ASS^
		001366491.2	001353420.1	4	1^ASS^	6^ASS^, 7^ASS^				
		001366492.2	001353421.1	5	1^ASS^	Δ2–3, 6^ASS^				
	Tspan18	130783.5	570139.3	1	1–3	3–9	9			
	Tspan19	001100917.2	001094387.1	1	1–2	2–9	9			
UPK1b	Tspan20	006952.4	008883.2	1	1–2	2–8	8			
UPK1a	Tspan21	007000.3	008931.1	1	1–2	2–9	9			
		001281443.1	001268372.1	2		+6			+6	+6
PRPH2	Tspan22	000322.5	000313.2	1	1	1–3	3			
ROM1	Tspan23	000327.3	000318.1	1	1	1–3	3			
CD151	Tspan24	004357.5	004348.2	1	1–3	3–9	9			
		139030.3	620599.1	1	Δ2					
		139029.1	620598.1	1	1^ASS^					
		001039490.1	001034579.1	1	1^ASS^ Δ2					
CD53	Tspan25	001040033.1	001035122.1	1	1–3	3–9	9			
		000560.4	000551.1	1	Δ1, 2^ASS^					
		001320638.1	001307567.1	2	Δ1, 2^ASS^	Δ6–7				
		from Natural Killer Cells cDNA		3	N/A	Δ5–8	N/A	N/A		
CD37	Tspan26	001774.3	001765.1	1	1	1–8	8			
		001040031.2	001035120.1	2		1^ASS^				
CD82	Tspan27	002231.4	002222.1	1	1–3	3–10	10			
		001024844.1	001020015.1	2		Δ6				
		from Human Brain cDNA		3	N/A	Δ9	N/A	N/A		
		Lee *et al*. 2003		4	N/A	Δ7	N/A	N/A		
CD81	Tspan28	004356.3	004347.1	1	1	1–8	8			
		001297649.1	001284578.1	2	Δ1	Δ1, 2^ASS^				
CD9	Tspan29	001769.4	001760.1	1	1	1–8	8			
		001330312.2	001317241.1	2	↔1	↔1		X		
CD63	Tspan30	001780.5	001771.1	1	1–2	2–8	8			
		001257389.1	001244318.1	1	↔1			X		
		001257390.1	001244319.1	1	↔1					
		001257391.1	001244320.1	1	Δ1	2^ASS^				
		001257392.1	001244321.1	2	Δ1	3^ASS^				
		001257400.1	001244329.1	3	↔1, Δ2	Δ2				
		001257401.1	001244330.1	3	↔1, Δ2	Δ2				
		001267698.1	001254627.1	1	1^ASS^					
	Tspan31	005981.5	005972.1	1	1	1–6	6			
		001330168.2	001317097.1	2		Δ2–3				
		001330169.2	001317098.1	3		1^ASS^				
	Tspan32	139022.2	620591.3	1	1	1–10	10			
	Tspan33	178562.5	848657.1	1	1	1–8	8	X		

Left, historic names used with priority over the systematic names in the NCBI database. More historic names with lower priority are listed in Table S1. Second and third columns, mRNA variants are sorted by systematic name, next sorted by the NCBI variant number for the mRNA. For Tpan16 and Tspan21, the first mRNAs variants are trispanins, and the second ones are tetraspanins. In these cases, we moved up the second mRNA variants referring to them as conventional tetraspanins (isoforms 1). Forth column, NCBI reference sequence number for protein (NP). Column 5 lists the isoform number. Column 6 lists the exons forming the 5′ UTR of the respective splice variant 1, being the reference sequence for comparisons with the 5′ UTR of the other alternatively spliced variants. For the alternatively spliced variants, the column lists the eliminated exon(s) (∆), the number of an exchanged exon (↔) or the number of an exon after which another exon has been introduced (+). In case an alternative splice site (ASS) is used, the number of the exon with the ASS is given. Columns 7 and 8 provide the same information for the ORF and the 3′ UTR, respectively. Column9; a cross indicates an open reading frame upstream of the ORF. Modifications by AS generating a new stop codon are shown in column 10, and modifications generating a frame shift in column 11. N/A, not available.

**Figure 2:**
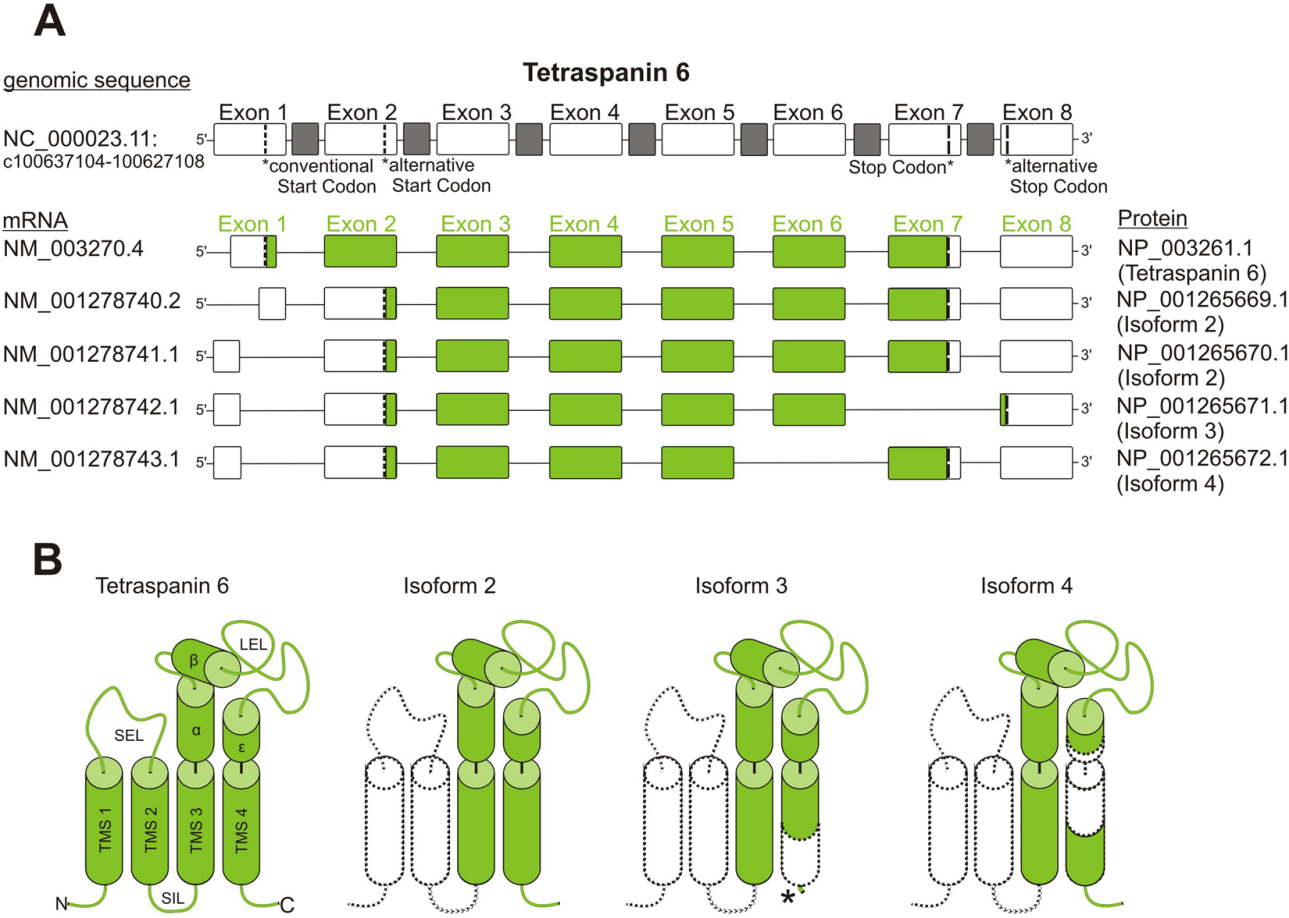
Isoforms of Tspan6. (**A**) Top, cartoon illustrating the genomic sequence of Tspan6 as exons (white boxes) and introns (grey boxes). Exon numbering refers to the genomic sequence. From the genomic sequence, five different mRNAs derive. Here, exon numbering (green) refers to mRNA variant 1. Green boxes mark the open reading frame. Exon-skipping is indicated by leaving out the exon. When compared to splice variant 1, a shortened exon box indicates the use of an AS site. The green exon numbering is used for comparison of the respective mRNA variant 1 to the splice variants in Tab. 1. (**B**) Left, helical structural elements of Tspan6 predicted by Seigneuret *et al*.^12^. The cartoons of the isoforms only illustrate the alterations in the primary structure and are no predictions of the protein topology. TMS, transmembrane segment; SIL, small intracellular loop; SEL, small extracellular loop; LEL, large extracellular loop; α - ε, helices of the LEL. Dashed lines mark missing parts (filled white). The asterisk in isoform 3 marks the alternative C-terminus.

Compared to the structure of a conventional tetraspanin (Fig. 1), non-conventional tetraspanins display broad structural variability. As examples, we explain the isoforms of Tspan6 (for illustration of isoforms for Tspan2, Tspan3, Tspan16, Tspan17, CD53, CD82, CD63, and Tspan31 see Figs S2–S9, respectively). Figure 2A shows the genomic sequence together with five mRNAs, from which four are derived by AS. In Fig. 2B, we depict the proteins deriving from the mRNA splice variants. Illustrated are remaining and deleted protein segments with reference to the conventional tetraspanin topology (Fig. 1), not yet predicting how the deletion may affect protein topology and/or the numbers of TMSs. Apart from the deletion of protein segments, in all Tspan6 splice variants AS produces additional changes in the 5′-UTR (untranslated region). These changes eventually cause diminished expression (see below).

The first mRNA codes for the conventional Tspan6 (isoform 1). In all other isoforms, the first two TMSs are missing (Fig. 2B). The second and third mRNA differs in their 5′-end but have the same alternative start codon. Therefore, both yield isoform 2 with large part of the N-terminus deleted, including TMS1, the SEL and TMS2. Also in case of isoform 3 this alternative start codon is used, resulting in TMS1/SEL/TMS2-deletion. Moreover, splicing eliminates exon 7 by which an alternative stop codon located in exon 8 is used. This causes deletion of the C-terminal half of TMS4 and an alternative C-terminus. Finally, isoform 4 again uses the alternative start codon, resulting in the N-terminal truncation. Moreover, exon 6 is eliminated, and thus the C-terminal end of the LEL ε-helix and the N-terminal half of TMS4 are not encoded.

We wondered whether such deletions also occur in other species and analyzed tetraspanin isoforms in mouse. Here, the database has lesser entries, as only 31 tetraspanins are described, four of them with provisional status only, and in general there are not that many mRNA variants available. Still, we identify eight non-conventional isoforms, including isoforms with only three predicted TMSs, LEL deletions, and changes in the N-terminus (Table S2). Between the two species, there is no direct correlation on the level of specific tetraspanins, but there is overlap in the type of structural change caused by AS. That not all structural variations occurring in human are also found in mouse maybe explained by the smaller data base and/or that the two species share only about a quarter of alternatively used exons^35,36^.

### Structural variability defines classes of non-conventional tetraspanins

**Figure 3:**
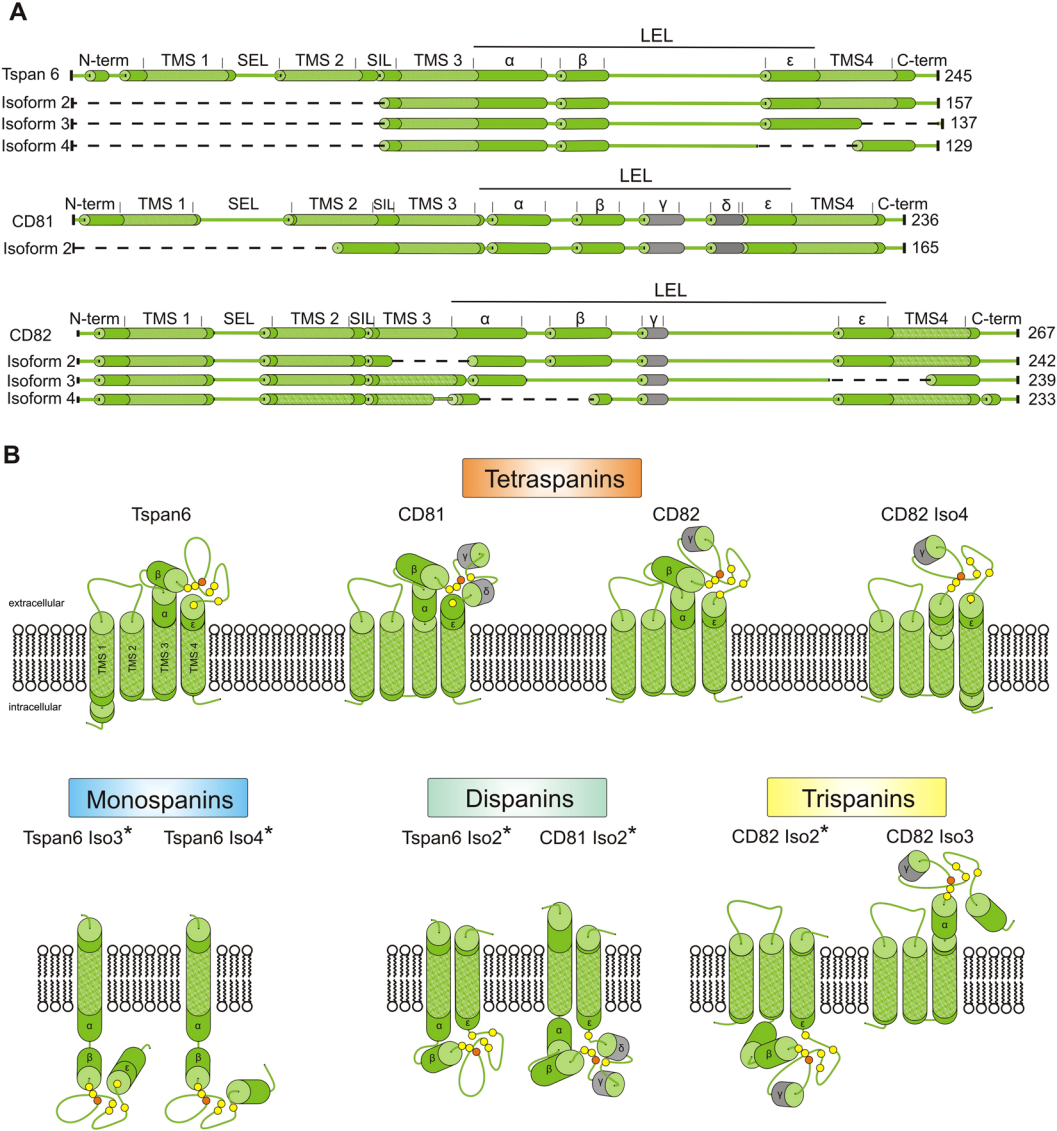
Predicted topology of tetraspanin isoforms. (**A**) Linearized proteins. Dashed lines indicate deleted parts. Green cylinders, α-helical structure predicted by Jpred. Grey cylinders, α-helical structure not predicted by Jpred but by Seigneuret *et al*.^12^, and in case of CD81 revealed from crystallographic data (Kitadokoru et al., 2001). Patterned green marks the predicted transmembrane helices (TMHMM Server, 2.0). The length of the sections scales with the number of amino acids. TMS, transmembrane segment; SEL, small extracellular loop; SIL, small intracellular loop; LEL, large extracellular loop; α - ε, α-helices in the LEL^11^. For Tspan6, no alpha helical structure of the variable domain is predicted wherefore no γ- and δ-helix are depicted. The AS of Tspan6 Iso3 leads to an alternative C-terminus. For CD82 in the variable domain only the γ-helix is predicted to be α-helical. (**B**) Topology of the tetraspanin isoforms illustrated in (**A**) with reference to the prediction which parts are intra- and extracellular (TMHMM Server, 2.0). Isoforms with an inverted topology are indicated by an asterisk. Yellow and orange spheres indicate cysteine- and glycine-residues, respectively. Cysteine-residues form disulfide bridges in the LEL; the glycine-residue is part of a conserved CCG-motif.

**Figure 4:**
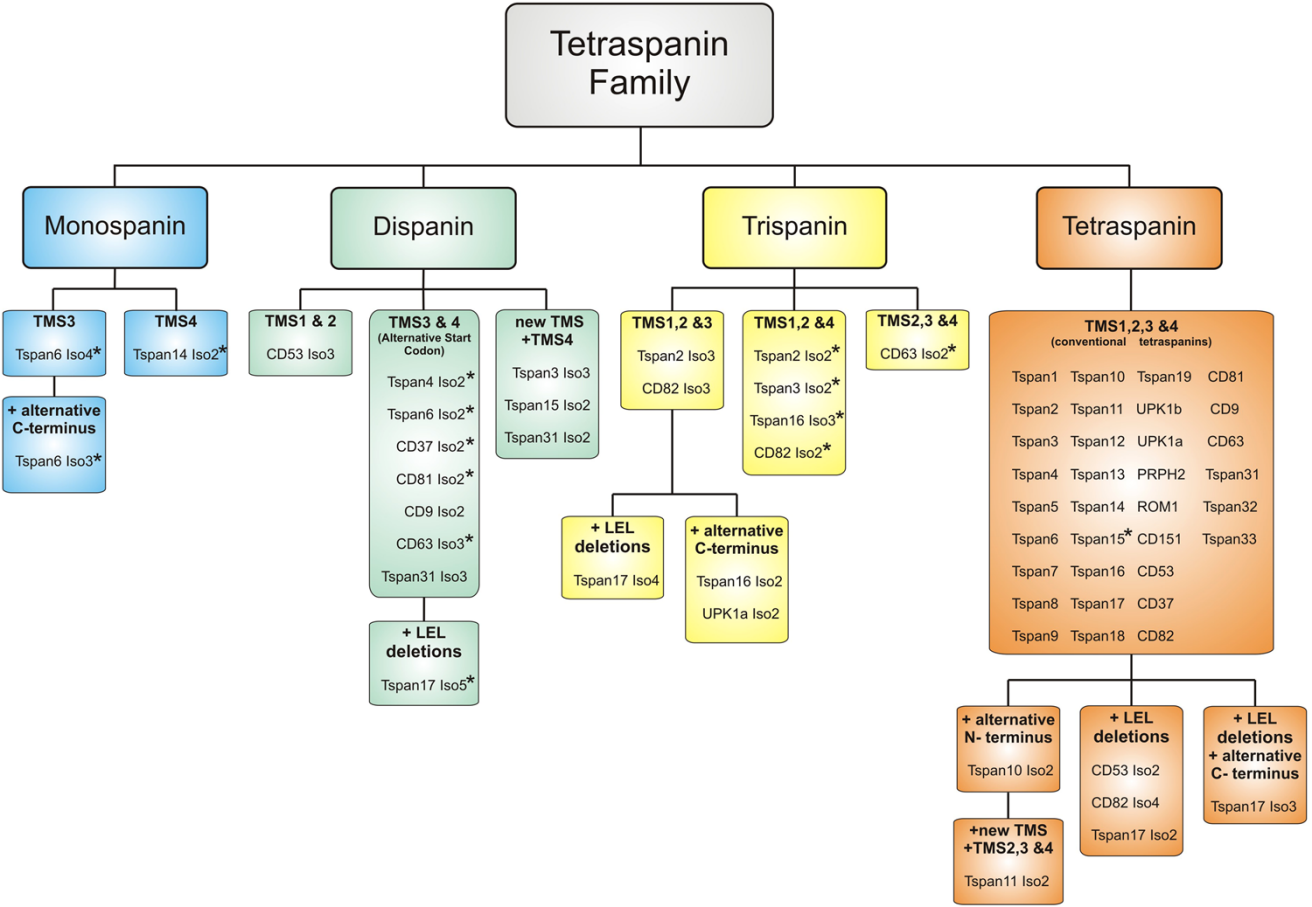
Classes of tetraspanins. Based on the analysis illustrated for the examples Tspan6, CD81 and CD82 (Fig. 3), all tetraspanins and their isoforms were classified as mono-, di-, tri- or tetraspanins. Subclasses result from the type of remaining TMSs, or whether a novel TMS is formed. Alteration of the N- or C- terminus, or the LEL define further subclasses. Isoforms with a partially or completely inverted topology are marked by an asterisk.

We next analyzed the topologies of the human non-conventional isoforms. Based on computational analyses of the proteins’ transmembrane helices (TMHMM Server, 2.0) we predict protein isoforms with overall one, two, three or four TMSs (Fig. 3). Thus, the isoforms categorize into four major classes, which are tetraspanins that structurally are mono-, di-, tri- and tetraspanins (Fig. 4). The monospan-tetraspanins maintain either TMS 3 or 4, and the dispan-tetraspanins TMSs 1 & 2, 3 & 4, or 4 and form a novel TMS. In the trispan-tetraspanins, any one of the TMSs is deleted, with the exception of TMS2. In one case in which TMS2 is remaining, TMS2 forms an extended TMS together with a half-deleted TMS1 (CD63 Iso2). Please note that for simplicity in the following we refer to e.g. trispan-tetraspanins just as trispanins.

In about half of the cases, AS results in a partially or completely inverted topology (indicated by an asterisk in Fig. 4). Surprisingly, an inverted topology is also predicted for the conventional Tspan15. However, experimental evidence indirectly indicates that murine Tspan15, which is also predicted to have an inverted topology, inserts with the correct topology^37^. Therefore, topology predictions should be treated with caution.

Most classes include representatives with a modified C-terminus. Moreover, in several cases AS affects the LEL, causing almost its complete elimination (CD53 Iso2), or shortening (CD82 Iso4 and Tspan17 Iso2, 3, 4 and 5). Based on the structure of the CD81 LEL^9^ and the prediction of secondary structural elements (Jpred 4.0), the short deletions would largely affect the variable domain of the LEL, which is interesting, as this part is supposed to encode the information for specific interactions. Finally, for Tspan10, the only tetraspanin with a large N-terminus, we find an isoform with a truncation in the large N-terminal domain (Tspan10 Iso2).

In summary, for most tetraspanins AS generates several mRNAs, yielding up to five isoforms per gene (see Tspan17). The number of non-conventional tetraspanin isoforms roughly equals the number of conventional tetraspanins. However, it is very likely that this is greatly underestimated as we included only validated/reviewed sequences. Moreover, the discovery of many yet undocumented sequences is expected.

### Expression of non-conventional tetraspanins

The question arises as to how likely the protein isoforms express at levels that would affect cellular function. Several factors would play a role, such as mRNA copy number (about which the data bank makes no statement), the stability of the mRNA, and the stability of the expressed protein.

In the following, we evaluate the stability of the 53 listed alternatively spliced mRNAs by analyzing features making mRNA prone to degradation or influencing its expression level (Table 1). All mRNAs lack retained introns and premature termination codons (PTCs), which would promote nonsense-mediated decay (NMD)^38^. This argues against enhanced degradation by such elements. For clarity, we have not included this information in Table 1.

**Figure 5:**
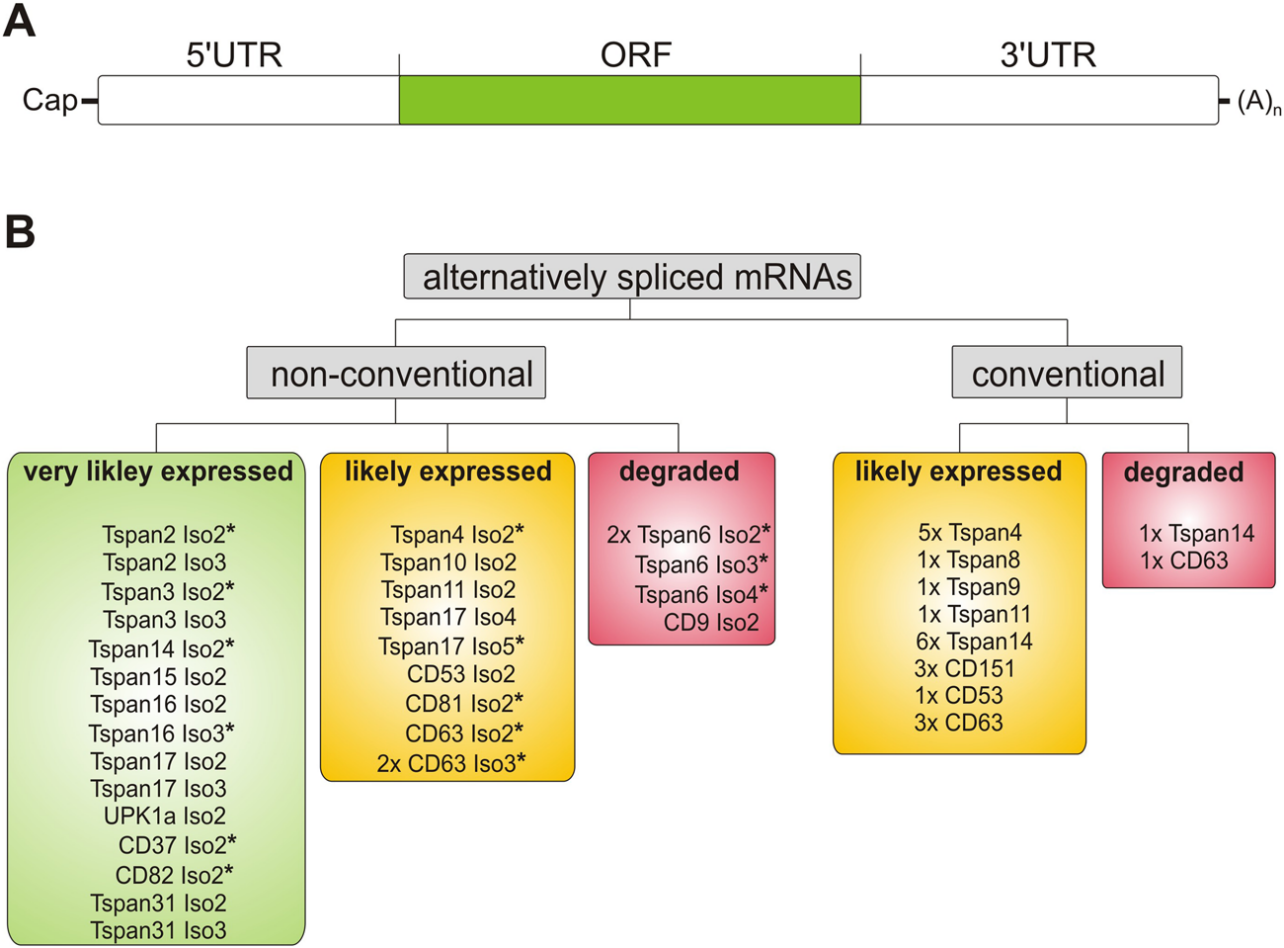
Evaluation of the expression probability of alternatively spliced mRNAs. (**A**) Sections of an mRNA. Cap, 5′-Cap; 5′ untranslated region (5′ UTR); ORF, open reading frame (ORF); 3′ untranslated region (3′ UTR); (A)_n_, poly(A) tail. (**B**) All alternatively spliced mRNAs lack retained introns and PTCs. In addition, mRNAs were analyzed for an upstream open reading frame (uORF), which induces NMD. They were also tested for alterations in the 3′UTR that could be associated with NMD, retention in the nucleus via nuclear RNA quality control, and miRNA-based gene silencing. Finally, they were analyzed for alteration in the 5′UTR that can alter the expression level of the mRNA. Based on these criteria, the mRNAs were sorted into three groups ranking their expression probability from very likely expressed (green - none of the criteria match), likely expressed (yellow - only alterations in the 5′UTR), or degraded (red - uORF and/or alteration in the 3′UTR).

We find 31 spliced mRNA variants with a sole alteration in the 5′ UTR (21 mRNAs coding for 8 conventional tetraspanins) or an alteration in the 5′ UTR and the ORF (10 mRNAs coding for 9 non-conventional tetraspanins). The 5′ UTR contains regulatory elements of translation. Effects on expression level upon alteration of this region are unpredictable^39–41^. To be on the safe side, we make a conservative estimate and assume that expression rather would be diminished. Therefore, the proteins for which these 31 mRNAs code for do not rate as being “very likely expressed”, but “likely expressed” (Fig. 5). The expression of two mRNAs coding for conventional and five mRNAs coding for non-conventional tetraspanins is not likely (Fig. 5). In these cases, we find alterations in the 3′ UTR that may cause NMD, retention in the nucleus and miRNA binding sites and/or an uORF (upstream open reading frame), which reduces expression levels 30–80% and/or makes the mRNA more likely subject to NMD^39,42^. However, this may be an overcautious rating as four not alternatively spliced mRNAs encoding conventional tetraspanins also contain uORFs, arguing against a complete uORF induced decay of tetraspanin mRNAs.

Finally, 15 mRNAs from 10 tetraspanin genes, all coding for non-conventional isoforms, are very likely expressed because they lack 5′ untranslated region (5′ UTR) alterations, uORF, PTCs or 3′ UTR alterations (Table 1) (Fig. 5). From these 15 mRNAs, nine code for proteins that have the extracellular loops on the extracellular site and therefore a membrane topology similar or identical to the respective conventional tetraspanin (Fig. 5), meaning their domains could in principle interact with binding partners. Moreover, there is another isoform with a shortened LEL (CD82 Iso4), from which the mRNA is unknown, wherefore we cannot evaluate its expression probability. Still, from published data we can safely conclude that this isoform expresses at levels that affect cellular function^43^.

In addition, for those isoforms amplified by PCR, we compared mRNA expression levels of the conventional to the non-conventional isoform(s) by quantitative real-time PCR. In the cDNA library from human brain, the Tspan15 Iso2 mRNA level is about 10% of the conventional form, and this wild-type Tspan15 is expressed at a several fold higher level than RPS9, encoding the 40S ribosomal protein S9, used as a reference (Table S3). Hence, although lower expressed than the conventional, the Tspan15 Iso2 expression level is substantial and in a dominant negative mechanism could be sufficient to alter cellular functions. Moreover, although expressed 10-fold less than Tspan15, Tspan15 Iso2 may dominate in the ER by accumulating there (compare Fig. S13).

CD53 expression in natural killer cells is also dominated by the conventional transcript, which is again found at a several fold higher level than RPS9. In comparison, CD53 Iso2 and Iso3 expression were found to be 5% and 3% of the conventional form. For CD53 Iso2 this is expected, as it has a lower mRNA expression probability when compared to Tspan15 Iso2 (Fig. 5). For CD53 Iso3 the expression probability cannot be evaluated, as only the coding sequence is known. In any case, for the CD53 isoforms it is difficult to predict whether they may influence cell physiology at such low expression levels. However, future analysis of other cDNA libraries may reveal cellular systems with higher expression levels.

### Retention in the ER of co-transported factors

**Figure 6:**
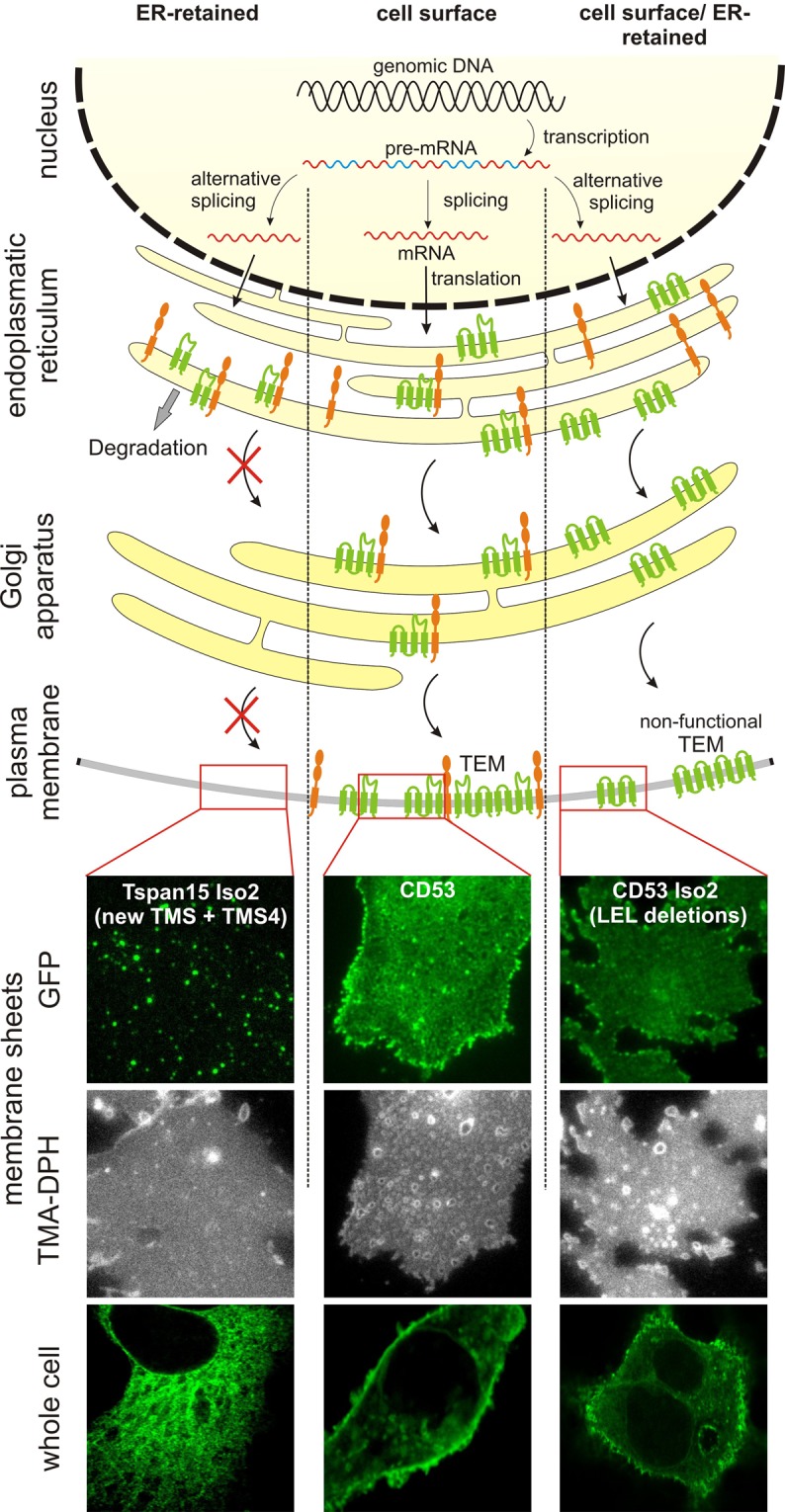
Trafficking and possible functions of non-conventional tetraspanins. Top, illustration of alternative splicing and trafficking from the ER to the plasma membrane. Transcription of the genomic DNA (black) generates pre-mRNA with introns (blue) and exons (red). AS generates two additional different mRNAs. After translation and insertion into the ER membrane, apart from the classical pathway (middle), isoforms may behave differently in two ways. Middle, the conventional tetraspanin (green) interacts with a binding partner (orange) and both are co-transported to the plasma membrane, where the tetraspanin forms a TEM. Left, most isoforms lack TMS. The isoform shown (green) is an example from the largest group of dispanins. They cannot exit the ER, but may still interact with other proteins. Thus, if it is degraded together with the binding partner, the surface expression level of the binding partner is altered. Right, the LEL deleted isoform (green) does not interact with its binding partner (orange) but exits the ER and forms TEMs in the plasma membrane. These TEMs would lack one or more co-factors and would therefore be non-functional or differently acting TEMs. Bottom, the lower panels show confocal micrographs of GFP-labeled Tspan15 Iso2 (the conventional Tspan15 is shown in Fig. S11), CD53 or CD53 Iso2 expressed in HepG2 cells (for non-GFP-expressing control cells see Fig. S10; Western blot analysis documents the correct size of the expressed constructs; see Fig. S12). Tspan15 reaches the plasma membrane (Fig. S11), whereas Tspan15 Iso2 remains in the ER (for co-staining analysis with an ER marker see Fig. S13). Bottom, upper panels, ER retention is confirmed by analysis of cell-free plasma membrane sheets that were visualized by the membrane dye TMA-DPH. In the respective GFP-channel, only a few Tspan15 Iso2 spots are detected, that arise from ER-PM contact sites^50^. In contrast, CD53 and CD53 Iso2 readily reach the plasma membrane, albeit CD53 Iso2 less efficient. CD53 Iso2 has lost its glycosylation sites and therefore appears in Western blot analysis as a single band (Fig. S12).

What might be the physiological effects of expressing non-conventional tetraspanins? In most cases described here, alternative splicing results in expression of variants with missing TMSs (compare Fig. 4). The role of the individual TMSs for proper folding has been studied to some extent, and especially tight packing of the TMSs1 and 2 appear to be crucial for proper tetraspanin folding^44^. Moreover, all four TMSs of Tspan20 are required for proper protein folding and forward-trafficking from the ER to the plasma membrane^45^. Thus, formation of a proper four-helix bundle structure appears to be crucial for ER exit. In conclusion, it appears to be very unlikely that tetraspanins with missing TMSs will be able to leave the ER. In fact, when studying the distribution of Tspan15 Iso2, which is a dispanin, we find retention in the ER (Fig. 6 and Fig. S13).

Yet, tetraspanin variants retained in the ER could affect cell physiology in two ways: First, complementation of a truncated tetraspanin via interaction with the “missing” helix of its full-length counterpart is possible, eventually resulting in improper folding of the full-length tetraspanin. Via a domino effect, this could result in cross-linked tetraspanins not leaving the ER. Actually, formation of unspecific tetraspanin aggregates has been suggested to be a mechanism causing ER retention^45^. Likely, these aggregates would be degraded and therefore such a mechanism would decrease the tetraspanin level at the cell-surface. Second, isoforms retained in the ER could still bind to their interaction partners, holding these in the ER and causing their degradation (Fig. 6). Evidence that such a mechanisms could exist comes from a study in which a mutation in the CD81 gene produces an isoform that is lacking TMS4, which is accompanied by a lack of expression of the CD81 interaction partner CD19^46^.

### Non-functional TEMs

While deletions of TMSs cause ER retention, modifications of the N- or C-terminus, or the LEL may still allow proteins to traffic to the cell membrane. Previously, it has been shown that deletions of segments in the CD81 LEL (deleting the α/β-, γ/δ-, γ- or δ-helical segment(s)) do not result in inefficient plasma membrane targeting^47^. Moreover, deletion of the entire LEL in CD53 Iso2 still allows for trafficking to the cell membrane (Fig. 6). In Jurkat T cells, that express endogenous CD81 at high levels, the additional expression of the CD81 mutant lacking the δ-helical segment inhibits viral uptake^47^, which indicates that the mutant has a dominant negative effect. This suggests that LEL deletion mutants might still be able to integrate into TEMs into which otherwise the conventional tetraspanin locates. However, as the deletion mutant does not properly interact anymore with its interaction partners, the TEM becomes non-functional (Fig. 6). It is also possible that the TEM loses only part of its functionality, resulting e.g. in aberrant cellular signaling.

## Conclusion

Little is known about the effect of AS on membrane proteins. Using the tetraspanin family as example, we studied whether AS enriches the gene products, revealing a large structural variability of tetraspanin isoforms. We speculate that non-conventional tetraspanins may regulate ER exit of tetraspanins and their interaction partners, form non-functional TEMs, or TEMs with different roles.

## Materials and Methods

### Sequence acquisition and cloning

The human tetraspanin sequences are acquired from the National Center for Biotechnology Information (NCBI) database for genes (as of 5^th^ June 2019), listed under ‘NCBI Reference Sequence (RefSeq) - mRNA and Protein(s)’. For human tetraspanins, we considered only sequences with the status report ‘reviewed’ or ‘validated’.

Two additional sequences were obtained by PCR from cDNA libraries (kindly provided by the AG Kolanus, LIMES institute, Bonn). The cDNA libraries used as PCR template were from human brain for Tspan15 Iso2 and CD82 Iso3, and from natural killer cells for CD53 Iso2 and CD53 Iso3. We employed Q5 High-Fidelity DNA Polymerase (NEB, # M0491S) and primers aligning with the 5′- and 3′-end of the ORF (without a stop codon) of the corresponding conventional tetraspanin. Primers carried a XhoI restriction site at the 5′- end, before the Kozak sequence and the start codon, and a blunt 3′-end. The following primers were used: Tspan15, 5′-TATTATCTCGAGCATGCCGCGCGGGGACTCGGAGC-3′ (fwd) and 5′-ATTGGGGTAGCACAAGCAGCATCCCG-3′ (rev); CD53, 5′-TATTATCTCGAGCATGGGCATGAGTAGCTTGAAAC-3′ (fwd) and 5′-TAGCCCTATGGTCTGGCTGG-3′ (rev); CD82 5′-TATTATCTCGAGCATGGGCTCAGCCTGTATCAAAGTC-3′ (fwd) and 5′-GTACTTGGGGACCTTGCTGTAGTCTTCGG-3′ (rev). The PCR products were digested with XhoI and the inserts were ligated into a pEGFP-C1 vector (Clontech, #6084-1) containing a monomeric enhanced GFP variant^47^. Also the backbone vector was amplified via PCR using the primers 5′-ATGGTGAGCAAGGGCGAGG-3′ (fwd) and 5′- ATAATACTCGAGGGATCTGACGGTTCACTAAACC- 3′ (rev). The amplified sequences were XhoI (NEB, #R0146S) digested and ligated using T4 Polynucleotide Kinase (NEB, #M0201S) and T4 DNA Ligase (NEB, #M0202S). The constructs were verified by sequencing (Eurofins GATC Biotech GmbH).

### Expression analysis by quantitative real-time PCR

Isoform-specific expression was measured in human cDNA samples from brain and natural killer cells by quantitative real-time PCR using the Maxima SYBR Green qPCR Master Mix (Thermo Scientific, #K0221). Primers used for intron-spanning assay: all CD53 isoforms, 5′-TCATGGTAGTTGCCTTCCTGG -3′ (fwd); CD53, 5′-CACATACTCATTCAGCTTCTGTTC-3′ (rev); CD53 Iso2, 5′-CGCATAGCAACCCTTCTGTTC-3′ (rev); CD53 Iso3, 5′-CATCCCCAACACCGACATAAG-3′ (rev); all Tspan15 isoforms, 5′-CTGCAGTCGTGGTACTGATTC-3′ (rev); Tspan15, 5′-TCCGGAACCAGACCATTGAC-3′ (fwd); Tspan15 Iso2, 5′-CCGTGTTCTGGACCATTGAC-3′ (fwd); RSP9, 5′-CTGCTGACGCTTGATGAGAA-3′ (fwd) and 5′-CAGCTTCATCTTGCCCTA-3′ (rev). Reactions were run in a total volume of 10 µl containing 200 pM of each primer with the following program: 40 cycles with incubation at 95 °C for 10 min, followed by 30 s at 60 °C and 30 s at 72 °C on a Roche LightCycler^®^ 480 II. The experiment was performed twice with each reaction pipetted in duplicates. Data were analyzed by advanced relative quantification in the LightCycler^®^ 480 Software using RPS9 as a reference.

### Analysis of transmembrane segments

The protein sequences were analyzed employing the program TMHMM Server v. 2.0 (http://www.cbs.dtu.dk/services/TMHMM/^48^). The program predicts with a certain likelihood the length and the position of transmembrane segments, and the intra- and extracellular localization of the segments connected to the TMSs. These TMSs lengths and the lengths of the interconnecting segments are shown in Table S1. In addition, for Tspan10, 19 and 22, we considered TMS as positively predicted if the segment had a total length of 15–35 residues. From these residues, the central 13–33 residues had a transmembrane probability ≥54%, and were flanked by one intracellular and one extracellular residue with a lower transmembrane probability^48^. In some cases, TMSs were shifted or shortened due to AS by a few amino acids. Here, we classified the TMS as a novel one if the shift was greater than five amino acids or more than 1/3 of the original TMS was replaced. Finally, the analysis indicates which domains or segments are changed by AS.

### Structure prediction

The helical structural elements of the isoforms shown in Fig. 3 were predicted combining the results from the TMS prediction by the program TMHMM Server v. 2.0 and a secondary structure prediction of the complete protein (JPred4; http://www.compbio.dundee.ac.uk/jpred4^49^). The relative lengths of the segments containing predicted helices, their position in the membrane, and the interconnecting segments were illustrated. The approximate position of the cysteine residues within the variable part of the LEL and the glycine and cysteine residues in the ubiquitously conserved cysteine-cysteine-glycine motif (CCG motifs)^12^ are also shown. The β-strands were not illustrated. The structural suggestions of the interconnecting domains refer to previous drawings of tetraspanins and claim no accuracy.

### Analysis of AS generated changes of mRNA

To determine the alterations by AS, we used the program BioEdit v7.0.5 (http://www.mbio.ncsu.edu/BioEdit/bioedit.html). Exons were defined with reference to the NCBI database. The changes generated by AS were identified by sequence alignment of the splice variant with the conventional tetraspanin sequence and are summarized in Table 1. Specifically, we tested for alterations in the 5′UTR and 3′UTR, NMD (nonsense mediated decay) initiating PTCs and uORFs (upstream open reading frames), which all may affect the mRNA expression level. The 5′UTR and 3′UTR alterations were directly extracted from the alignment described above. The PTCs were tested for their NMD initiation potential by measuring the distance between the PTC and the most 3′ exon-exon junction (EEJ). PTCs more than 50 nucleotides upstream of the most 3′-EEJ were categorized as NMD promoting. However, none of the PTCs fulfilled this criterion. The sequences were also tested for uORFs by translating them to their corresponding amino acid sequence (ExPASy Bioinformatics Resource Portal; https://web.expasy.org/translate/) and testing them for an in frame open reading frame 5′ upstream of the tetraspanin start codon.

### Expression and imaging of GFP-labelled tetraspanins

HepG2 cells were transfected essentially as described^47^ with a vector for expression of GFP (pEGFP-N1, clonetech, #6085-1) or the above described vectors for expression of GFP fused to the C-terminus of Tspan15, Tspan15 Iso2, CD53 or CD53 Iso2. Cell-free membrane sheets were produced by short ultra-sound pulses^47^. If not stated otherwise, epi-fluorescence microscopy was employed for imaging membrane sheets and whole cells that in this case additionally were visualized with the membrane dye TMA-DPH (Invitrogen, #T204). TMA-DPH and GFP-fluorescence were imaged by epi-fluorescence microscopy essentially as described^47^. For confocal microscopy, cells additionally expressed KDEL-RFP and were stained as described below. They were imaged in the confocal mode of a 4-channel easy3D superresolution STED optics module (Abberior Instruments) coupled to an Olympus IX83 confocal microscope (Olympus, Tokyo, Japan), equipped with an UPlanSApo 100x (1.4 NA) objective (Olympus, Tokyo, Japan). For imaging details see below. Additionally, GFP was excited with a 485 nm laser and recorded with a 525/50 nm filter.

### Western blotting of GFP-tagged Tetraspanins

HepG2 cells were lysed 22 h after transfection by addition of buffer A (82.25 mM Tris-HCl, 32.9% (w/v) glycerol, 2.6% SDS, pH 6.8). The lysate was mixed 1:4 with buffer A with additional 5% β-mercaptoethanol and was heated to 95 °C for 10 min. Proteins were separated on a 12% SDS-PAGE and blotted on a Nitrocellulose membrane (Carl Roth, #HP40.1) using a tank blot system (Bio-Rad, #1703930). The membrane was blocked with blocking buffer, a 1:1 mixture of TBS-T (50 mM Tris, 150 mM NaCl, 0.05% Tween 20 (w/v), pH 7.4) and Odyssey Blocking Buffer (Li-Cor, #927-40000). Membranes were incubated with primary antibodies in blocking buffer and incubated over night at 4 °C. Primary antibodies used were rabbit polyclonal anti-GFP (Thermo Fisher Scientific, #A-11122) diluted 1:2,000 and mouse monoclonal anti-beta-Actin (Cell Signaling, #3700) diluted 1:5,000. The secondary antibodies donkey anti-mouse coupled to IRDye 680RD (Li-Cor, #926-68072) and donkey anti-rabbit coupled to IRDye 800CW (Li-Cor, #926-32213) were diluted 1:10,000 in blocking buffer. The membrane was imaged using an Odyssey Classic Imaging System (Li-Cor).

### Colocalization of Tetraspanin 15 with the endoplasmic reticulum

HepG2 cells were transfected with GFP-labelled Tspan15 or Tspan15 Iso2 as described above. Six hours after transfection, cells were transduced with a KDEL-RFP fusion construct (BacMam 2.0, Life Technologies, # C10591) specifically targeting the ER according to the manufacturer’s instructions with 20 particles per cell for an additional 16 h. Cells were fixed with 4% paraformaldehyde (PFA) in PBS for 30 minutes. Fixation solution was removed and residual PFA was quenched with 50 mM NH_4_Cl in PBS for 30 minutes. Cells were then permeabilized with 0.2% Triton X-100 in PBS for 2 minutes and blocked with 3% BSA for 1 hour at room temperature (RT). To enhance the GFP- and RFP-signal, samples were incubated with GFP-Booster Atto647N (Chromotek, # gba647n) and RFP-Booster Atto594 (Chromotek, # rba594) diluted 1:200 in 1% BSA for 1 hour at RT. At last, samples were washed with PBS and mounted onto microscopy slides with ProLong® Gold antifade mounting medium (Invitrogen, #P36930). Coverslips were cured for 24 hours and sealed with nail polish. Cells were imaged in the confocal mode of the superresolution STED microscope described above. Atto594/RFP was excited with a 561 laser and detected with a 580–630 nm filter (red channel). Atto647N was excited with a 640 nm laser and recorded with a 650–720 nm filter (long red channel). For all images, pixel size was set to 50 nm and pinhole size was set to 60 µm.

Colocalization analysis was performed with the program ImageJ. Regions of interest (ROIs) were placed into the red channel to an area that showed the typical ER network structure, and then propagated to the long red channel (illustrated in the figure employing a green lookup table). The Pearson correlation coefficient (PCC) between the two areas marked by the ROIs was calculated with a custom made ImageJ macro.

## Supplementary information

Supplementary Tables and Figures
