# Supplementary material for: Classes of non-conventional tetraspanins defined by alternative splicing
Source: Sci Rep. 2019 Oct 1;9:14075. doi: 10.1038/s41598-019-50267-0 (PMC6773723; doi:10.1038/s41598-019-50267-0)
Supplement: Supplementary file 1 — Supplementary Tables and Figures [file 41598_2019_50267_MOESM1_ESM.docx]

Classes of non-conventional tetraspanins defined by alternative splicing

Nikolas Hochheimer^1^, Ricarda Sies^1^, Anna C. Aschenbrenner^2,3^, Dirk Schneider^4^ and Thorsten Lang^1,*^

^1^Department of Membrane Biochemistry and ^2^Department of Genomics and Immunoregulation, Life & Medical Sciences Institute (LIMES), University of Bonn, Carl-Troll-Straße 31, 53115 Bonn, Germany, ^3^Department of Internal Medicine and Radboud Center for Infectious Diseases (RCI), Radboud University Nijmegen Medical Center, Geert Grooteplein Zuid 8, 6525 GA Nijmegen, The Netherlands,  ^4^Institute of Pharmacy and Biochemistry, Johannes Gutenberg University Mainz, Johann-Joachim-Becher-Weg 30, 55128 Mainz, Germany

*correspondence should be addressed to thorsten.lang@uni-bonn.de

**Supplementary Tables S1 – S3 and Figures S1 – S13**

| Name | | | | Length of Tetraspanin Domains | | | | | | | | |  |  |
| --- | --- | --- | --- | --- | --- | --- | --- | --- | --- | --- | --- | --- | --- | --- |
| historic names | | | systematic | N-term | TMS1 | SEL | TMS2 | SIL | TMS3 | LEL | TMS4 | C-term | full length | LEL-Cys |
|  | | |  |  |  |  |  |  |  |  |  |  |  |  |
| NET-1, TM4C | | | Tspan1 | 11 | 23 | 19 | 23 | 12 | 23 | 103 | 23 | 4 | 241 | 6 |
| NET-3, TSN2 | | | **Tspan2** | 12 | 23 | 19 | 23 | 11 | 23 | 76 | 23 | 11 | 221 | 4 |
| TM4-A, TM4SF8 | | | **Tspan3** | 12 | 23 | 14 | 23 | 12 | 23 | 104 | 23 | 19 | 253 | 6 |
| NAG-2, TM4SF7 | | | **Tspan4** | 11 | 23 | 19 | 23 | 6 | 23 | 96 | 23 | 14 | 238 | 6 |
| NET-4, TM4SF9 | | | Tspan5 | 19 | 23 | 19 | 23 | 8 | 23 | 119 | 23 | 11 | 268 | 8 |
| T245, TM4SF6 | | | **Tspan6** | 19 | 23 | 14 | 23 | 12 | 23 | 96 | 23 | 12 | 245 | 6 |
| A15, CCG-B7 | | | Tspan7 | 16 | 23 | 19 | 20 | 11 | 23 | 102 | 23 | 12 | 249 | 6 |
| CO-029, TM4SF3 | | | Tspan8 | 12 | 23 | 14 | 23 | 12 | 23 | 99 | 23 | 8 | 237 | 6 |
| NET-5, PP1057 | | | Tspan9 | 11 | 23 | 19 | 23 | 6 | 23 | 99 | 23 | 12 | 239 | 6 |
| OCSP | | | **Tspan10** | 116 | 23 | 19 | 23 | 12 | 23 | 116 | 20 | 41 | 393 | 8 |
| VSSW1971 | | | **Tspan11** | 20 | 23 | 14 | 23 | 12 | 23 | 106 | 23 | 9 | 253 | 6 |
| EVR5, NET-2 | | | Tspan12 | 12 | 23 | 19 | 23 | 12 | 23 | 115 | 23 | 55 | 305 | 6 |
| NET-6, TM4SF13 | | | Tspan13 | 12 | 23 | 9 | 23 | 6 | 20 | 71 | 23 | 17 | 204 | 6 (+2) |
| DC-TM4F2, TM4SF14 | | | **Tspan14** | 19 | 23 | 19 | 23 | 6 | 23 | 119 | 23 | 15 | 270 | 8 |
| 2700063A19Rik, NET-7 | | | **Tspan15** | 21 | 23 | 12 | 23 | 14 | 23 | 118 | 23 | 37 | 294 | 8 |
| TM-8, TM4-B | | | **Tspan16** | 12 | 23 | 19 | 23 | 11 | 23 | 107 | 23 | 3 | 244 | 6 |
| FBX23, TM4SF17 | | | **Tspan17** | 19 | 23 | 21 | 23 | 8 | 23 | 120 | 23 | 72 | 332 | 8 |
| TSPAN | | | Tspan18 | 12 | 23 | 14 | 23 | 12 | 23 | 114 | 23 | 4 | 248 | 6 |
|  | | | Tspan19 | 11 | 23 | 21 | 23 | 6 | 23 | 106 | 17 | 18 | 248 | 6 |
| UPK1b, UPIB, UPK1 | | | Tspan20 | 12 | 23 | 19 | 23 | 11 | 23 | 121 | 23 | 5 | 260 | 6 |
| UPK1a, UP1A, UPIA | | | **Tspan21** | 12 | 23 | 24 | 23 | 11 | 23 | 116 | 23 | 3 | 258 | 6 |
| PRPH2, AOFMD, RDS | | | Tspan22 | 20 | 23 | 18 | 17 | 21 | 23 | 130 | 23 | 71 | 346 | 6 (+1) |
| ROM1, ROSP1, RP7 | | | Tspan23 | 20 | 23 | 17 | 23 | 19 | 23 | 138 | 23 | 65 | 351 | 6 (+1) |
| CD151, GP27, PETA-3 | | | Tspan24 | 19 | 23 | 14 | 23 | 12 | 23 | 107 | 23 | 9 | 253 | 6 |
| CD53, MOX44 | | | **Tspan25** | 12 | 23 | 14 | 23 | 11 | 23 | 75 | 23 | 15 | 219 | 4 |
| CD37, GP52-40 | | | **Tspan26** | 12 | 26 | 19 | 23 | 6 | 23 | 134 | 23 | 15 | 281 | 6 |
| CD82, 4F9, KAI1 | | | **Tspan27** | 12 | 23 | 19 | 23 | 6 | 23 | 123 | 23 | 15 | 267 | 6 |
| CD81, CVID6, TAPA1 | | | **Tspan28** | 12 | 23 | 26 | 23 | 6 | 23 | 90 | 23 | 10 | 236 | 4 |
| CD9, BTCC-1, DRAP-27 | | | **Tspan29** | 12 | 23 | 24 | 23 | 6 | 23 | 83 | 23 | 11 | 228 | 4 |
| CD63, LAMP-3, ME491 | | | **Tspan30** | 12 | 23 | 14 | 23 | 12 | 23 | 98 | 23 | 10 | 238 | 6 |
| SAS |  | | **Tspan31** | 11 | 23 | 9 | 23 | 6 | 20 | 81 | 20 | 17 | 210 | 6 (+2) |
| ART1, TSSC6 | | | Tspan32 | 19 | 23 | 17 | 23 | 6 | 23 | 89 | 23 | 97 | 320 | 4 |
| PEN | | | Tspan33 | 24 | 23 | 14 | 23 | 12 | 23 | 119 | 23 | 22 | 283 | 8 |
|  | | | | | | | | | | | | | | |
|  | | Mean | | 17.8 | 23.1 | 17.3 | 22.7 | 10.1 | 22.8 | 105.8 | 22.6 | 22.4 | 264.6 | 6.1 |
|  | | Min | | 11 | 23 | 9 | 17 | 6 | 20 | 71 | 17 | 3 | 204 | 4 |
|  | | Max | | 116 | 26 | 26 | 23 | 21 | 23 | 138 | 23 | 97 | 393 | 8 |
|  | | | |  |  |  |  |  |  |  |  |  |  |  |

**Table S1**: Segment lengths of conventional tetraspanins. Listed are historic and systematic names. The historic names include at least two examples from the NCBI database listed under “Aliases”. In case historic names are used with priority over the systematic names, up to three historic names are shown. The tetraspanins with isoforms are written bold. The length of the protein domains are based on the TMHMM2.0 TMS predictions using the NCBI tetraspanin sequences. For the LEL, the number of disulfide bond forming cysteines is indicated together with the cysteines from the CCG motif (written in brackets). Bottom; mean, minimal and maximal values for each column.

| historic  Name | systematic Name | mRNA (NM_) | Protein (NP_) | Isoform | 5’UTR | ^ORF Exon^ | 3‘UTR | Class | Changes |
| --- | --- | --- | --- | --- | --- | --- | --- | --- | --- |
|  | Tspan1* | 133681.4 | 598442.1 | 1 | 1 - 2 | 2 - 8 | 8 |  |  |
|  | Tspan2 | 027533.3 | 081809.2 | 1 | 1 | 1 - 8 | 8 |  |  |
|  |  | 001243132.1 | 001230061.1 | 2 | ↔1 | ↔1 |  | Tetraspanin | alt. N-term, new TMS1, SEL shortened |
|  | Tspan3 | 019793.3 | 062767.3 | 1 | 1 | 1 - 7 | 7 |  |  |
|  | Tspan4 | 053082.3 | 444312.1 | 1 | 1 - 2 | 2 - 8 | 8 |  |  |
|  |  | 001252588.1 | 001239517.1 | 1 | ↔1 |  |  |  |  |
|  | Tspan5 | 019571.5 | 062517.1 | 1 | 1 | 1 - 8 | 8 |  |  |
|  | Tspan6 | 019656.3 | 062630.2 | 1 | 1 | 1 - 7 | 7 - 8 |  |  |
|  | Tspan7 | 019634.2 | 062608.2 | 1 | 1 | 1 - 7 | 7 - 8 |  |  |
|  | Tspan8 | 146010.2 | 666122.1 | 1 | 1 | 1 - 8 | 8 |  |  |
|  |  | 001168679.1 | 001162150.1 | 1 | 1^ASS^ |  |  |  |  |
|  |  | 001168680.1 | 001162151.1 | 1 | 1^ASS^ |  |  |  |  |
|  | Tspan9 | 175414.5 | 780623.1 | 1 | 1 - 3 | 3 - 9 | 9 |  |  |
|  |  | 001356301.1 | 001343230.1 | 1 | Δ2 |  |  |  |  |
|  | Tspan10** | 145363.2 | 663338.2 | 1 | 1 | 1 - 3 | 3 |  |  |
|  | Tspan11 | 026743.3 | 081019.1 | 1 | 1 - 2 | 2 - 8 | 8 |  |  |
|  | Tspan12 | 173007.4 | 766595.1 | 1 | 1 - 3 | 3 - 9 | 9 |  |  |
|  |  | 001363814.1 | 001350743.1 | 2 | Δ1 | Δ8 |  | Tetraspanin | LEL deletions |
|  | Tspan13* | 025359.3 | 079635.1 | 1 | 1 | 1 - 6 | 6 |  |  |
|  | Tspan14 | 145928.2 | 666040.1 | 1 | 1 - 2 | 2 - 9 | 9 |  |  |
|  |  | 001316748.1 | 001303677.1 | 1 | ↔1 |  |  |  |  |
|  | Tspan15 | 197996.2 | 932113.2 | 1 | 1 | 1 - 8 | 8 |  |  |
|  | Tspan16 | N/A | N/A | N/A |  |  |  |  |  |
|  | Tspan17 | 028841.3 | 083117.2 | 1 | 1 | 1 - 8 | 8 |  |  |
|  | Tspan18 | 183180.2 | 899003.1 | 1 | 1 - 5 | 5 - 10 | 10 |  |  |
|  | Tspan19 | N/A | N/A | N/A |  |  |  |  |  |
| UPK1b | Tspan20 | 178924.4 | 849255.2 | 1 | 1 - 2 | 2 - 8 | 8 |  |  |
| UPK1a | Tspan21* | 026815.2 | 081091.1 | 1 | 1 - 2 | 2 - 8 | 8 |  |  |
| PRPH2 | Tspan22 | 008938.2 | 032964.1 | 1 | 1 | 1 - 3 | 3 |  |  |
| ROM1 | Tspan23 | 009073.4 | 033099.3 | 1 | 1 | 1 - 3 | 3 |  |  |
| CD151 | Tspan24 | 009842.3 | 033972.2 | 1 | 1 - 3 | 3 - 8 | 8 |  |  |
|  |  | 001111049.1 | 001104519.1 | 1 | 1^ASS^ |  |  |  |  |
|  |  | 001111050.1 | 001104520.1 | 1 | ↔1, Δ2 |  |  |  |  |
| CD53 | Tspan25 | 007651.3 | 031677.1 | 1 | 1 - 2 | 2 - 8 | 8 |  |  |
| CD37 | Tspan26 | 001290802.1 | 001277731.1 | 1 | 1 | 1 - 9 | 9 |  |  |
|  |  | 001290804.1 | 001277733.1 | 2 |  | Δ2 |  | Tetraspanin | alt. N-term -TMS1- SEL-TMS2 |
|  |  | 007645.4 | 031671.1 | 3 | Δ1, 2^ASS^ | Δ1, 2^ASS^ |  | Tetraspanin | alt. N-term |
| CD82 | Tspan27 | 007656.5 | 031682.1 | 1 | 1 - 3 | 3 - 10 | 10 |  |  |
|  |  | 001136055.2 | 001129527.1 | 1 | ↔1 |  |  |  |  |
|  |  | 001271432.1 | 001258361.1 | 1 | +1 |  |  |  |  |
|  |  | 001271430.1 | 001258359.1 | 1 | Δ2 |  |  |  |  |
|  |  | 001271462.1 | 001258391.1 | 1 | ↔1 |  |  |  |  |
|  |  | 001271461.1 | 001258390.1 | 1 | ↔1 |  |  |  |  |
|  |  | 001271431.1 | 001258360.1 | 1 | ↔1, Δ2 |  |  |  |  |
| CD81 | Tspan28 | 133655.2 | 598416.1 | 1 | 1 | 1 - 8 | 8 |  |  |
| CD9 | Tspan29 | 007657.4 | 031683.1 | 1 | 1 | 1 - 8 | 8 |  |  |
| CD63 | Tspan30 | 001042580.1 | 001036045.1 | 1 | 1 - 2 | 2 - 8 | 8 |  |  |
|  |  | 007653.3 | 031679.1 | 1 | ↔1 |  |  |  |  |
|  |  | 001282966.1 | 001269895.1 | 1 | ↔1 |  |  |  |  |
|  | Tspan31* | 025982.4 | 080258.1 | 1 | 1 | 1 - 6 | 6 |  |  |
|  | Tspan32 | 001128080.2 | 001121552.1 | 1 | 1 | 1 - 9 | 9 |  |  |
|  |  | 020286.3 | 064682.1 | 2 | Δ1 | Δ1, 2^ASS^ |  | Trispanin | Δ N-term - TMS1 |
|  |  | 001128081.1 | 001121553.1 | 3 | Δ1 | Δ1, 2^ASS^, Δ7 |  | Trispanin | Δ N-term - TMS1, LEL deletion, new TMS4 |
|  |  | 001128082.1 | 001121554.1 | 4 | Δ1 | Δ1, 2^ASS^, Δ5, Δ7 |  | Trispanin | Δ N-term - TMS1, LEL deletion, new TMS4 |
|  | Tspan33 | 146173.3 | 666285.1 | 1 | 1 | 1 - 8 | 8 |  |  |
|  |  | 001301407.1 | 001288336.1 | 2 |  | 3^ASS^ |  | Tetraspanin | Deletion in SEL |

**Table S2.** *Tetraspanin splice variants in mouse.*

Mouse tetraspanin sequences are from the National Center for Biotechnology Information (NCBI) database for genes (as of 28^h^ August 2019). Left, historic names, second column systematic names (asterisk indicates that the status in the database is provisional and not reviewed or validated). Third column, mRNA variants are sorted by the NCBI variant number. Forth column, NCBI reference sequence number for protein (NP). Column 5 lists the isoform number. Column 6 lists the exons forming the 5’ UTR of the respective splice variant 1 (grey), being the reference sequence for comparisons with the 5’ UTR of the other alternatively spliced variants. For the alternatively spliced variants (white), the column gives the number of an eliminated exon (∆), the number of an exchanged exon (↔) or the number of an exon after which another exon has been introduced (+). In case an alternative splice site (ASS) is used, the number of the exon with the ASS is given. Columns 7 and 8 provide the same information for the ORF and the 3’ UTR, respectively. For the eight non-conventional isoforms, columns 9 indicates the class and column 10 lists the specific changes. **Conventional tetraspanin is predicted to have only three TMSs.

| Target | Target  Mean Cp | Reference | Reference  Mean Cp | Mean Target/Reference | ^SD^ | Mean  Ratio over conventional | SD |
| --- | --- | --- | --- | --- | --- | --- | --- |
| Tspan15 | 19.89 |  |  | 4.96 | 1.86 | 1.00 |  |
|  |  | RPS9 | 22.08 |  |  |  |  |
| Tspan15 Iso2 | 23.43 |  |  | 0.41 | 0.11 | 0.09 | 0.02 |
| CD53 | 18.34 |  |  | 4.79 | 1.30 | 1.00 |  |
|  |  | RPS9 | 20.54 |  |  |  |  |
| CD53 Iso2 | 22.55 |  |  | 0.25 | 0.05 | 0.05 | 0.01 |
| CD53 Iso3 | 23.66 |  |  | 0.12 | 0.04 | 0.03 | 0.00 |

**Table S3.** *Isoform-specific expression of CD53 and Tspan15.*

Listed are mean Cp (crossing point) values for expression of Tspan15 and CD53 isoforms in human brain and natural killer cells, respectively, as well as the mean target/reference ratios. “Ratio over conventional” compares isoform transcript levels to the transcript of each gene. Values are given as means ± SD (n = 4).

**CD53 Iso3 sequence from natural killer cell cDNA**

5’‑ ATGGGCATGAGTAGCTTGAAACTGCTGAAGTATGTCCTGTTTTTCTTCAACTTGCTCTTTTGGATCTGTGGCTGCTGCATTTTGGGCTTTGGGATCTACCTGCTGATCCACAACAACTTCGGAGTGCTCTTCCATAACCTCCCCTCCCTCACGCTGGGCAATGTGTTTGTCATCGTGGGCTCTATTATCATGGTAGTTGCCTTCCTGGGCTGCATGGGCTCTATCAAGGAAAACAAGTGTCTGCTTATGTCGGTGTTGGGGATGTCCTTTGCACTGACCCTGAACTGCCAGATTGACAAAACCAGCCAGACCATAGGGCTA -3’

**CD82 Iso3 sequence from human brain cDNA**

5’‑ ATGGGCTCAGCCTGTATCAAAGTCACCAAATACTTTCTCTTCCTCTTCAACTTGATCTTCTTTATCCTGGGCGCAGTGATCCTGGGCTTCGGGGTGTGGATCCTGGCCGACAAGAGCAGTTTCATCTCTGTCCTGCAAACCTCCTCCAGCTCGCTTAGGATGGGGGCCTATGTCTTCATCGGCGTGGGGGCAGTCACTATGCTCATGGGCTTCCTGGGCTGCATCGGCGCCGTCAACGAGGTCCGCTGCCTGCTGGGGCTGTACTTTGCTTTCCTGCTCCTGATCCTCATTGCCCAGGTGACGGCCGGGGCCCTCTTCTACTTCAACATGGGCAAGCTGAAGCAGGAGATGGGTGGCATCGTGACTGAGCTCATTCGAGACTACAACAGCAGTCGCGAGGACAGCCTGCAGGATGCCTGGGACTACGTGCAGGCTCAGGTGAAGTGCTGCGGCTGGGTCAGCTTCTACAACTGGACAGACAACGCTGAGCTCATGAATCGCCCTGAGGTCACCTACCCCTGTTCCTGCGAAGTCAAGGGGGAAGAGGACAACAGCCTTTCTGTGAGGAAGGGCTTCTGCGAGGCCCCCGGCAACAGGACCCAGAGTGGCAACCACCCTGAGGACTGGCCTGTGTACCAGGAGCTCCTGGGGATGGTCCTGTCCATCTGCTTGTGCCGGCACGTCCATTCCGAAGACTACAGCAAGGTCCCCAAGTAC -3’

**Figure S1**: *Nucleotide sequences isolated from cDNA libraries for CD53 Iso3 and CD82 Iso3.*


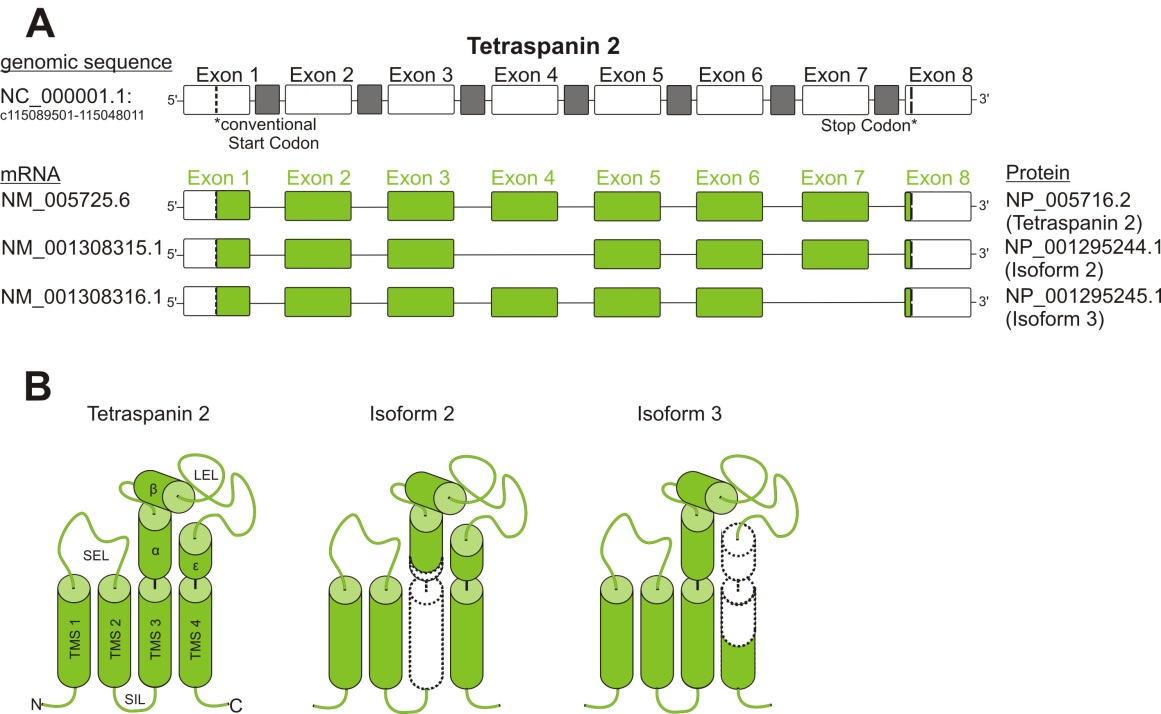


**Figure S2**: *Tspan2 isoforms.*

For scheme explanation, please see legend to figure 2.


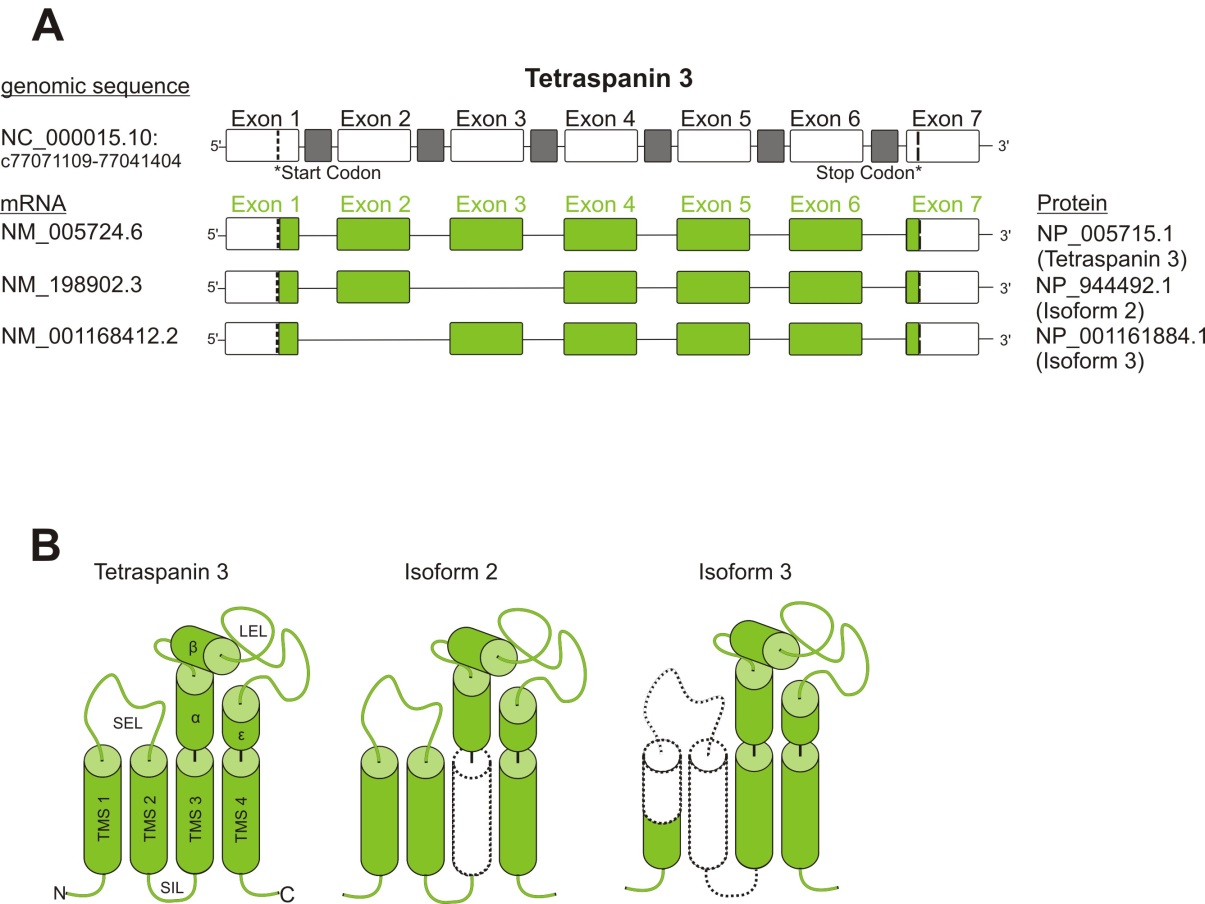


**Figure S3**: *Tspan3 isoforms.*

For scheme explanation, please see legend to figure 2.


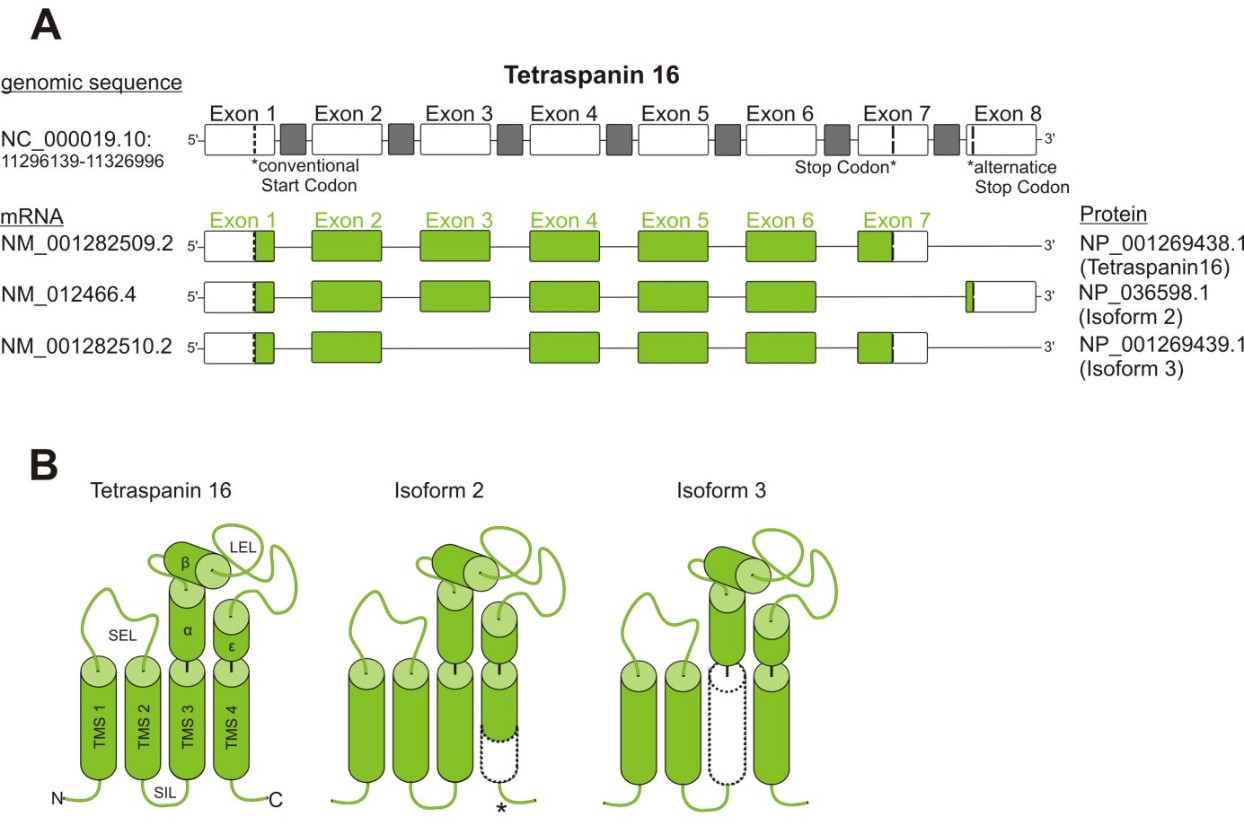


**Figure S4**: *Tspan16 isoforms.*

For scheme explanation, please see legend to figure 2. Asterisk indicates alternative C-terminus.


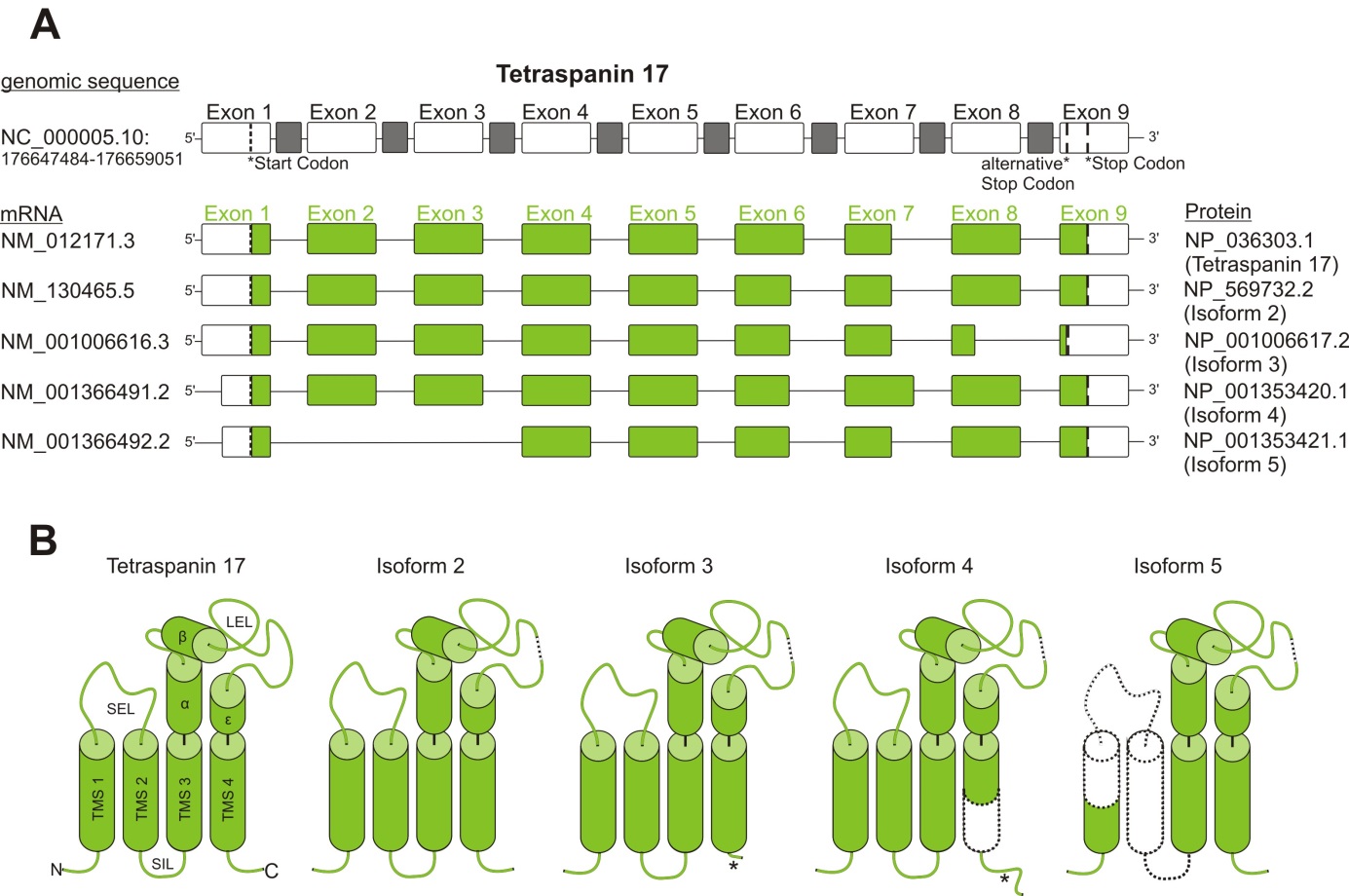


**Figure S5**: *Tspan17 Isoforms.*

For scheme explanation, please see legend to figure 2. Asterisk indicates alternative C-terminus.


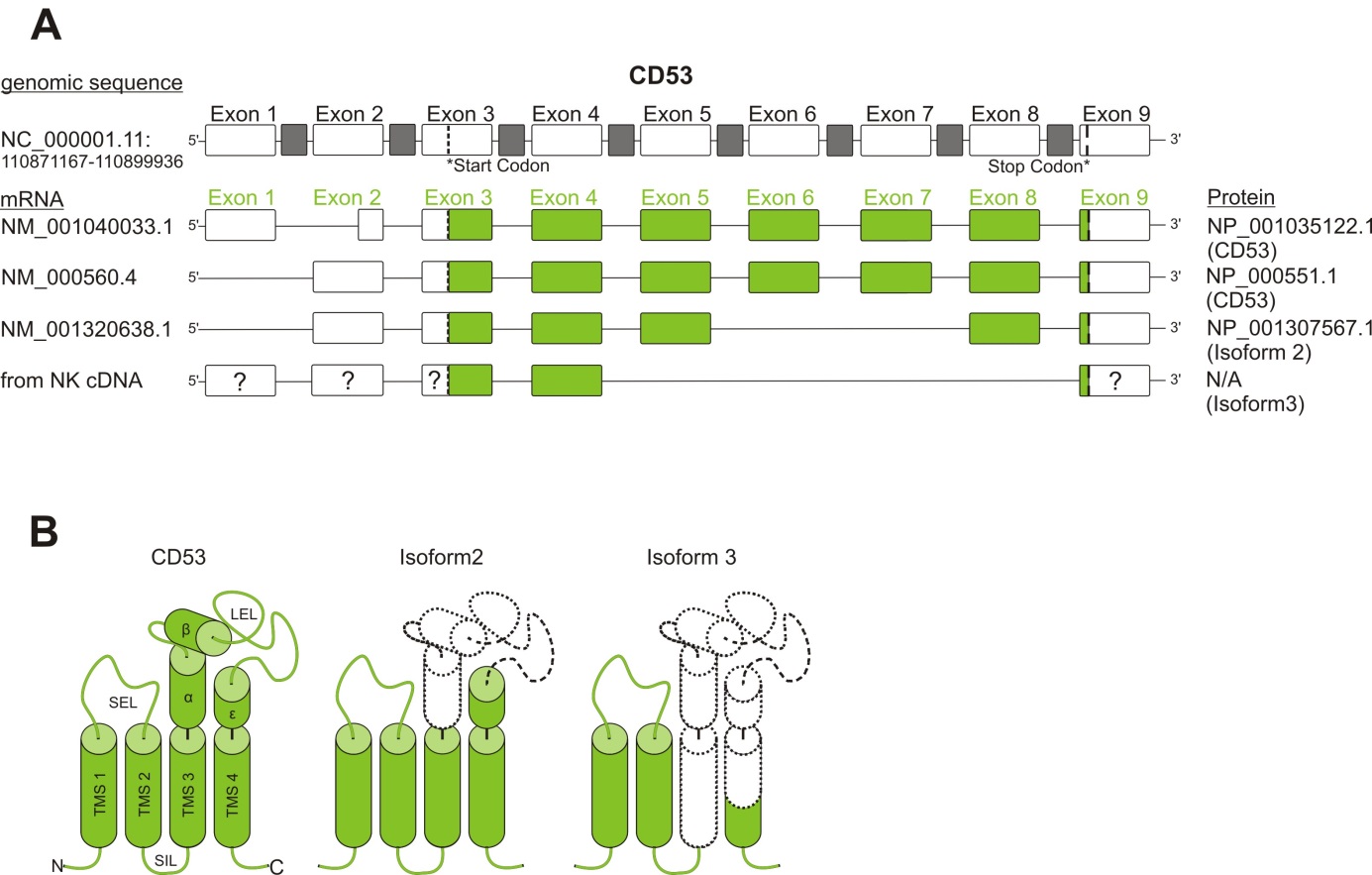


**Figure S6**: *CD53 isoforms.*

For scheme explanation, please see legend to figure 2. Please note that for isoform 3 the sequence of the untranslated regions is unknown.


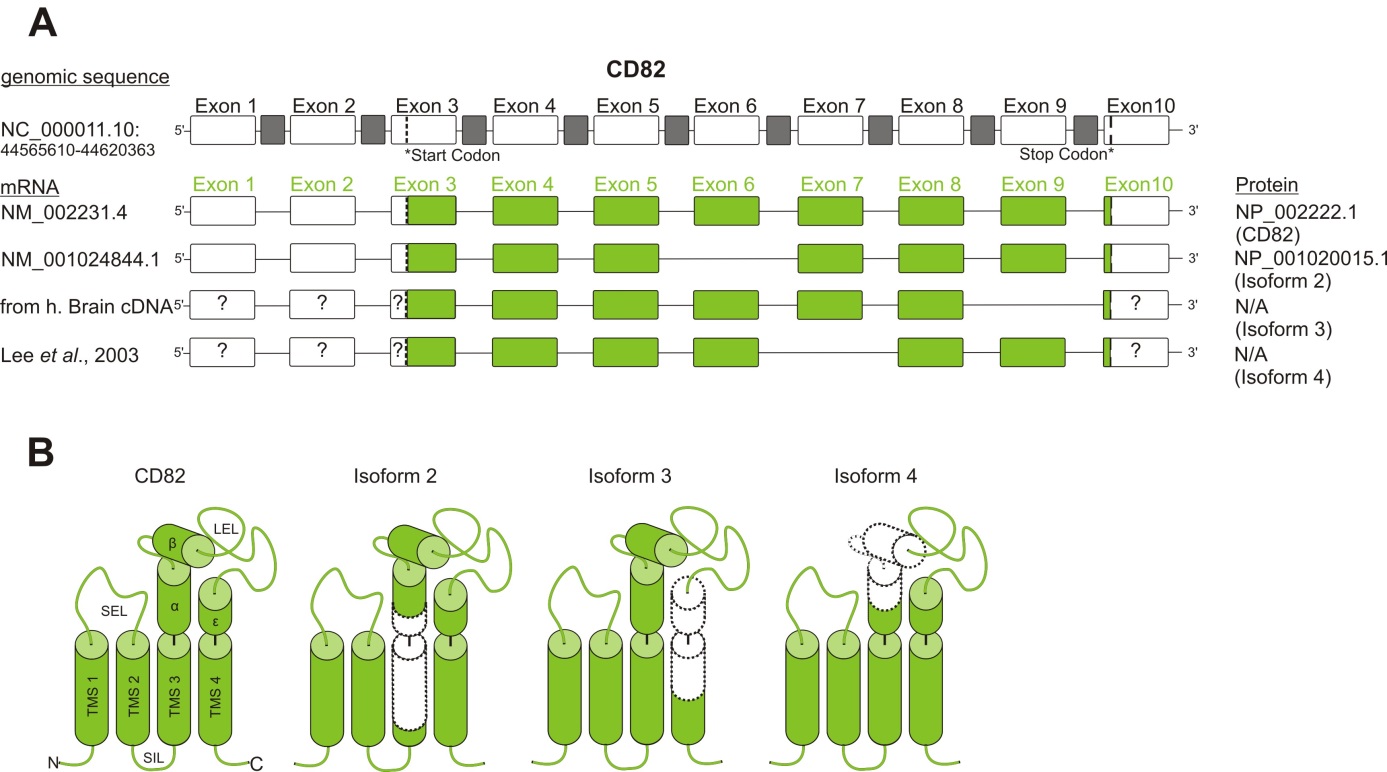


**Figure S7**: *CD82 Isoforms.*

For scheme explanation, please see legend to figure 2. Please note that for isoform 3 and 4 the sequence of the untranslated regions is unknown.


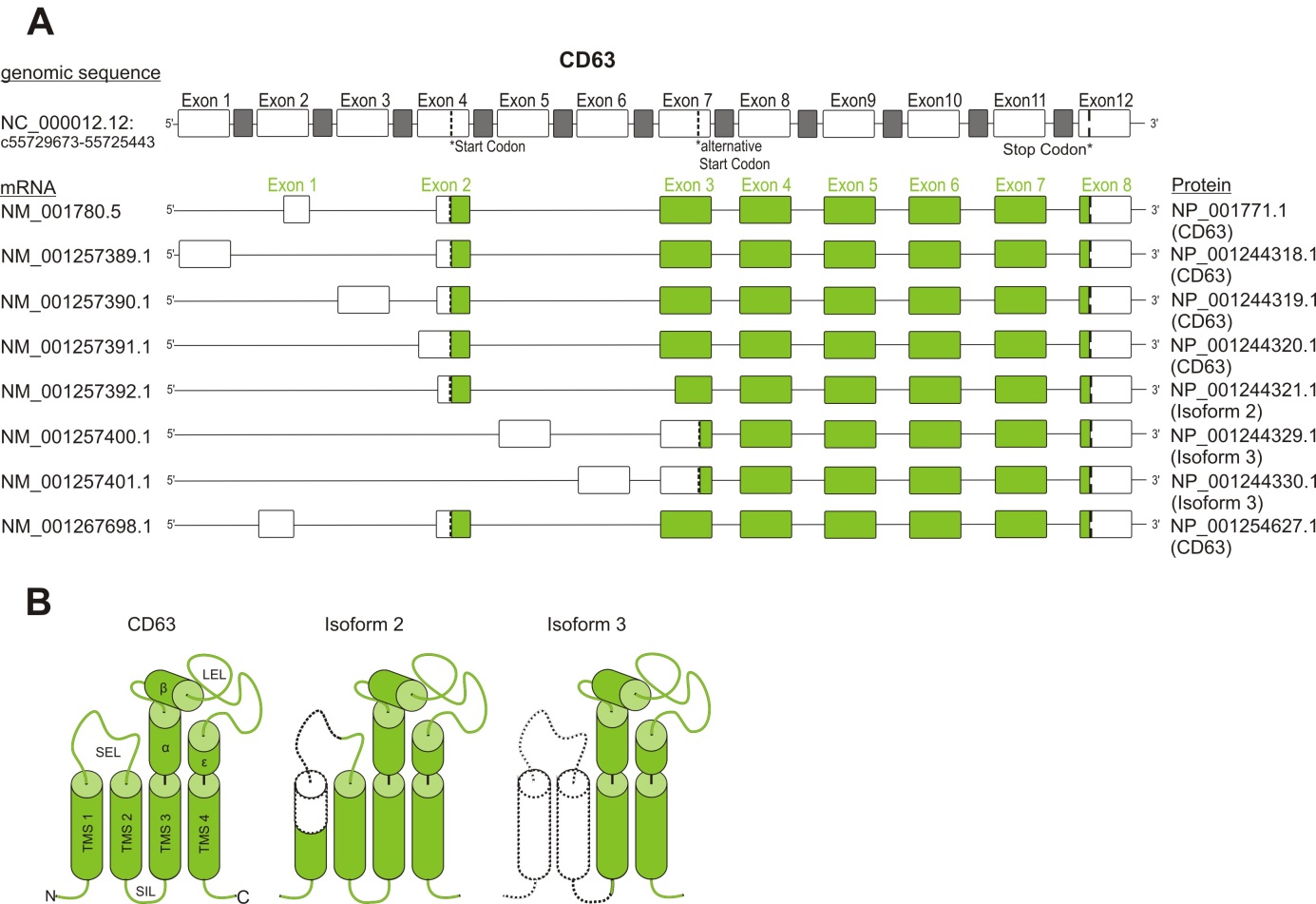


**Figure S8**: *CD63 Isoforms.*

For scheme explanation, please see legend to figure 2.


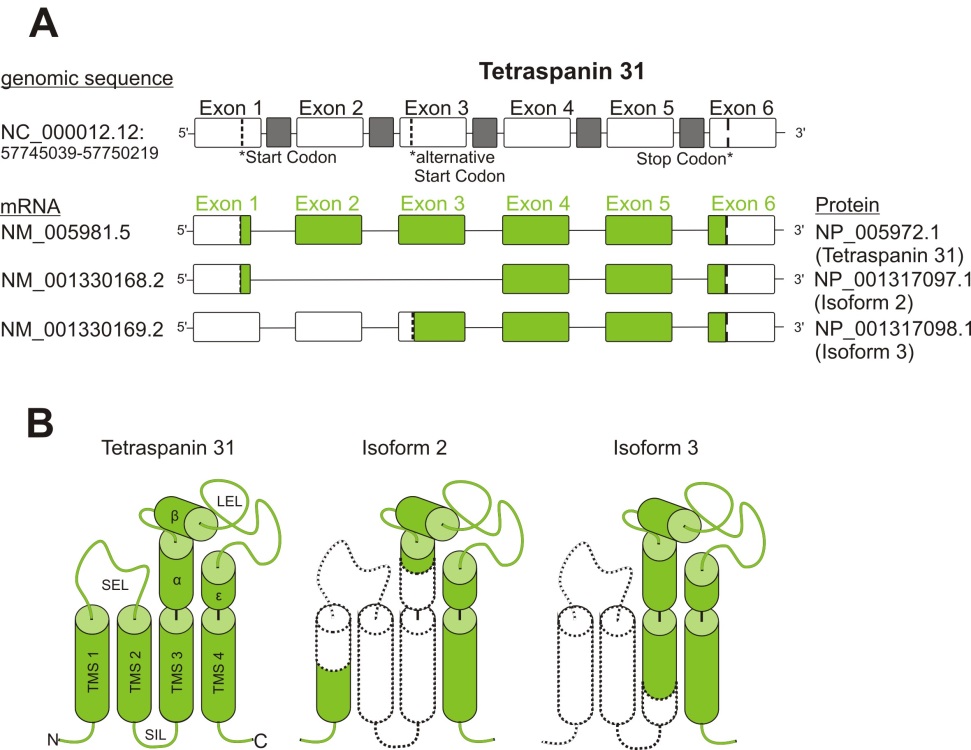


**Figure S9**: *Tspan31 Isoforms.*

For scheme explanation, please see legend to figure 2.


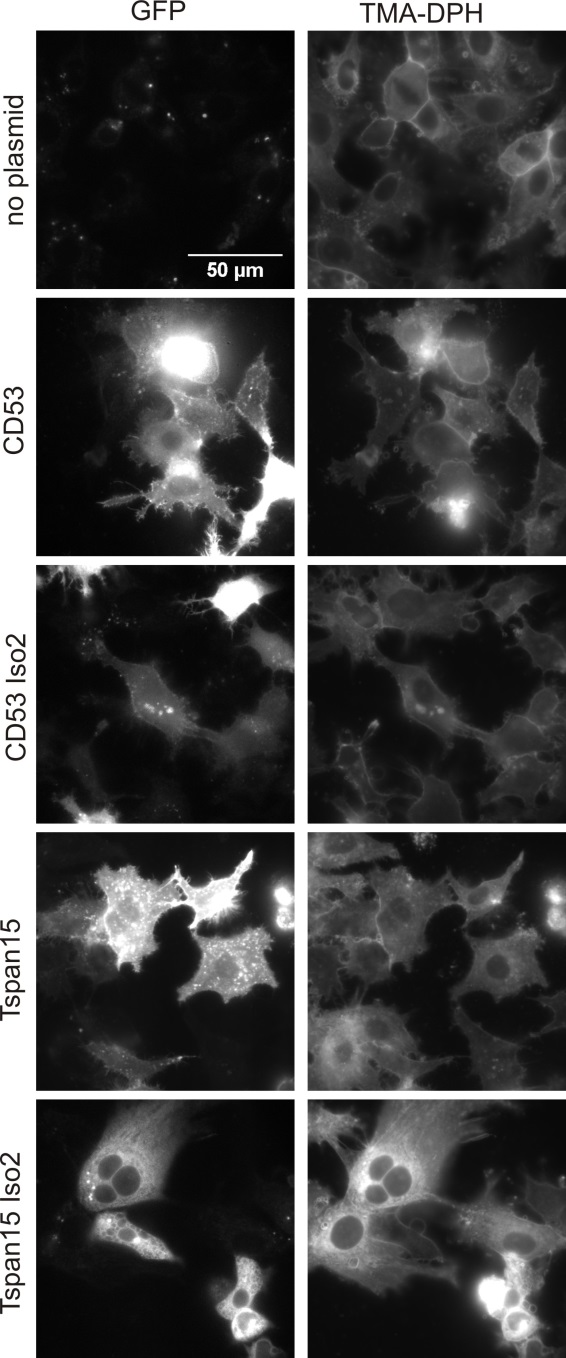


**Figure S10**: *Intensity of GFP signal in comparison to untransfected cells.*

HepG2 cells were transfected as described in the methods section without plasmid or plasmids coding for the indicated GFP-labelled tetraspanins. After 22 h cells were visualized by the membrane dye TMA-DPH and imaged in the green (left, GFP signal) and the blue (right, TMA-DPH fluorescence) channel. For evaluation of the GFP intensity, images in the green channel are scaled identically. The blue channel indicates the presence of cells. Comparison to the green channel indicates that not all cells are expressing the respective GFP-construct.


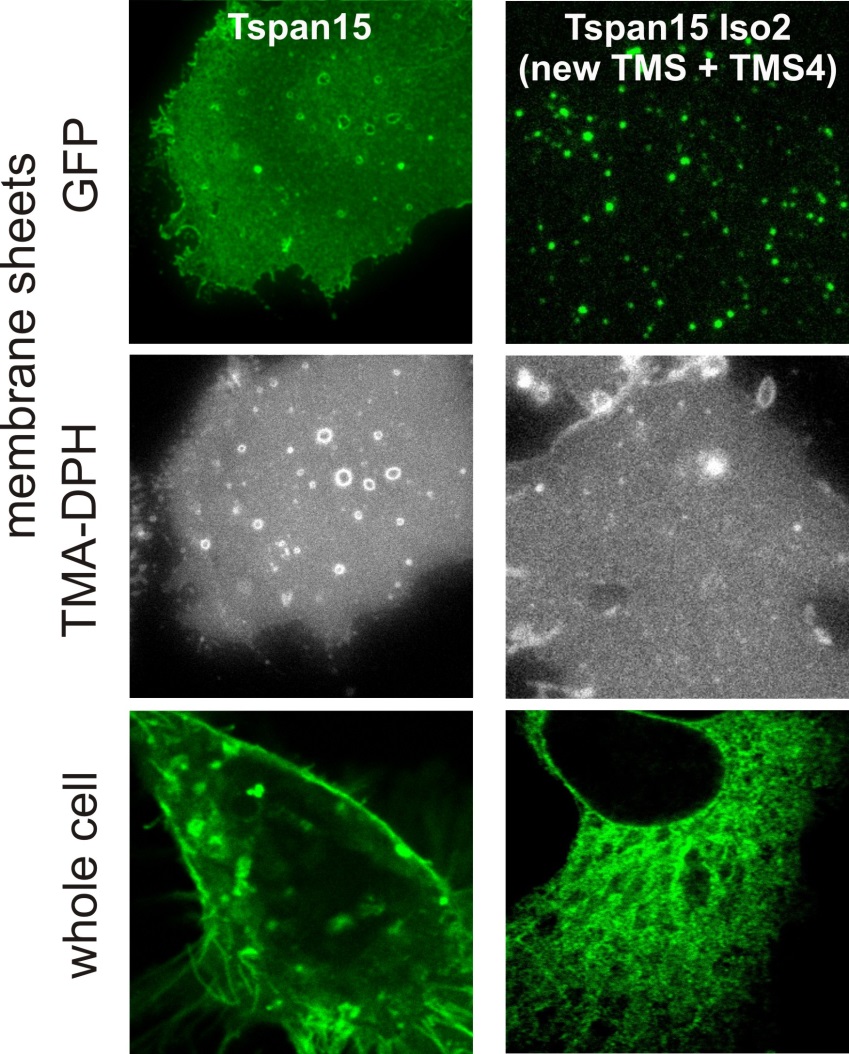


**Figure S11**: *Cellular distribution of Tspan15.*

Extension of Figure 6, illustrating a HepG2 cell expressing GFP-labelled conventional Tspan15 and a membrane sheet generated from a HepG2 cell expressing Tspan15-GFP. For comparison to Tspan15 Iso2, again the respective panels from Figure 6 are shown. For details, please see legend of Figure 6.


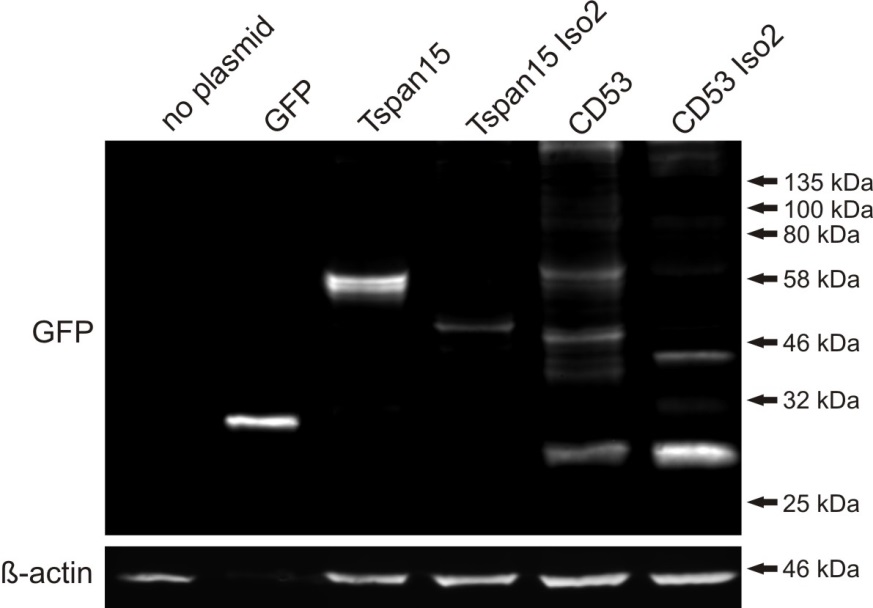


**Figure S12.** *Expression of GFP-labeled tetraspanins*

Top, HepG2 cells were transfected with no plasmid, a plasmid for GFP expression (≈ 27 kDa), or for expression of GFP-labelled conventional Tspan15 (≈ 60 kDa), Tspan15 Iso2 (≈ 51 kDa), conventional CD53 (≈ 51 kDa) or CD53 Iso2 (≈ 45 kDa). In case of GFP expression, the amount of loaded material was only 1/8 as otherwise the GFP signal in the Western Blot would be too strong. Please note that the conventional forms appear as double bands, which is due to glycosylation. In case of CD53, glycosylation causes a strong shift > 10 kDa toward higher apparent molecular mass, already described in the literature^1,2^. In contrast, CD53 Iso2 appears as a single band, as the glycosylation sites are deleted. CD53/CD53 Iso2 degradation products are of lower mass than GFP, indicating that they are not fluorescent and consequently not visualized in fluorescence microscopy. See Figure S14 for the full-length blot. Bottom, on the same membrane we immunoblotted for actin as a loading standard, visualized in a different channel. See Figure S15 for the full-length blots.


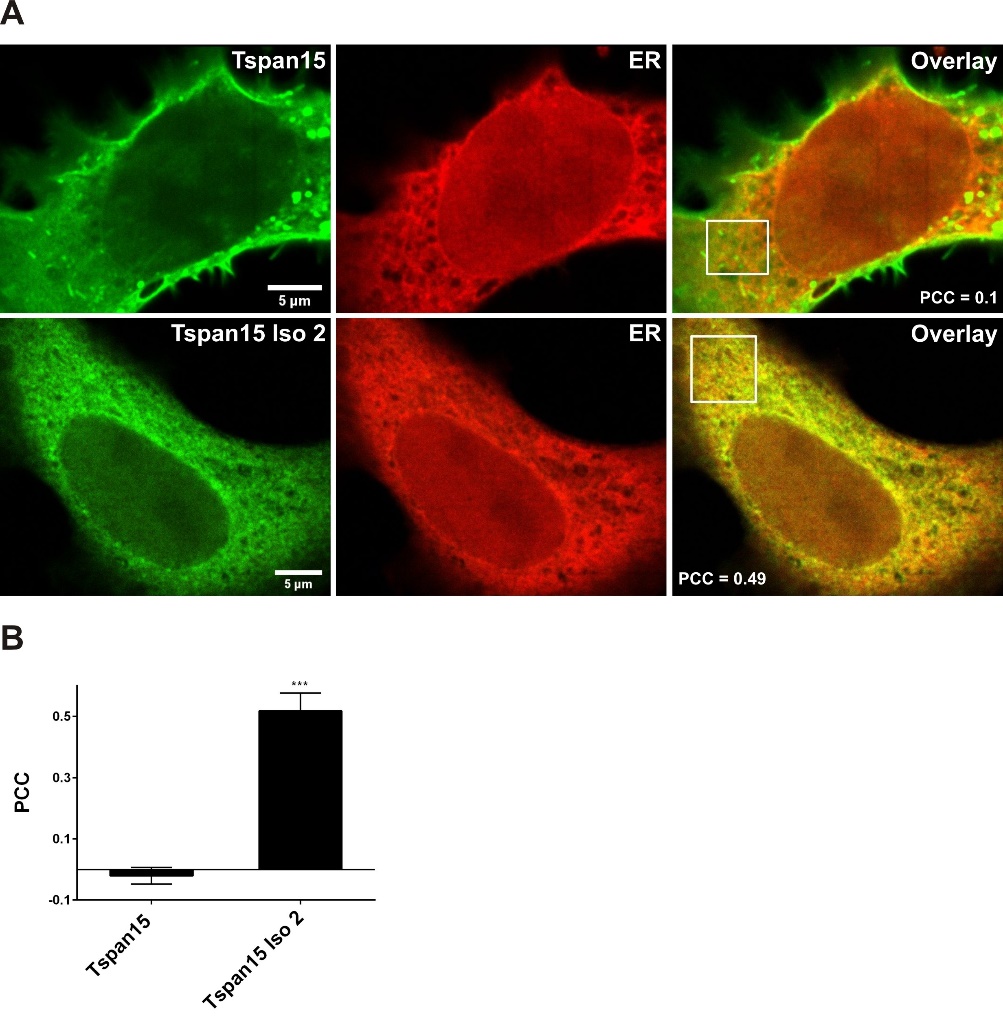


**Figure S13.** *Colocalization of Tspan15 and Tspan15 Iso2 with the ER.*

(A) Confocal micrographs showing equatorial sections of HepG2 cells expressing GFP-labelled conventional Tspan15 or Tspan15 Iso2 (shown in the green channel) and the ER marker KDEL-RFP (red channel). White boxes mark regions of interest (ROIs), in which colocalization between the two channels was measured by calculation of the Pearson correlation coefficient (PCC). For the shown cells, PCC values of 0.1 and 0.49 were obtained for Tspan15/ER and Tspan15 Iso2/ER, respectively (B) Average Pearson correlation coefficient. Values are given as means ± SEM (n = 4 biological replicates, for each replicate the values from 5 – 20 cells were averaged). Unpaired student’s t-test (***, p < 0.001).


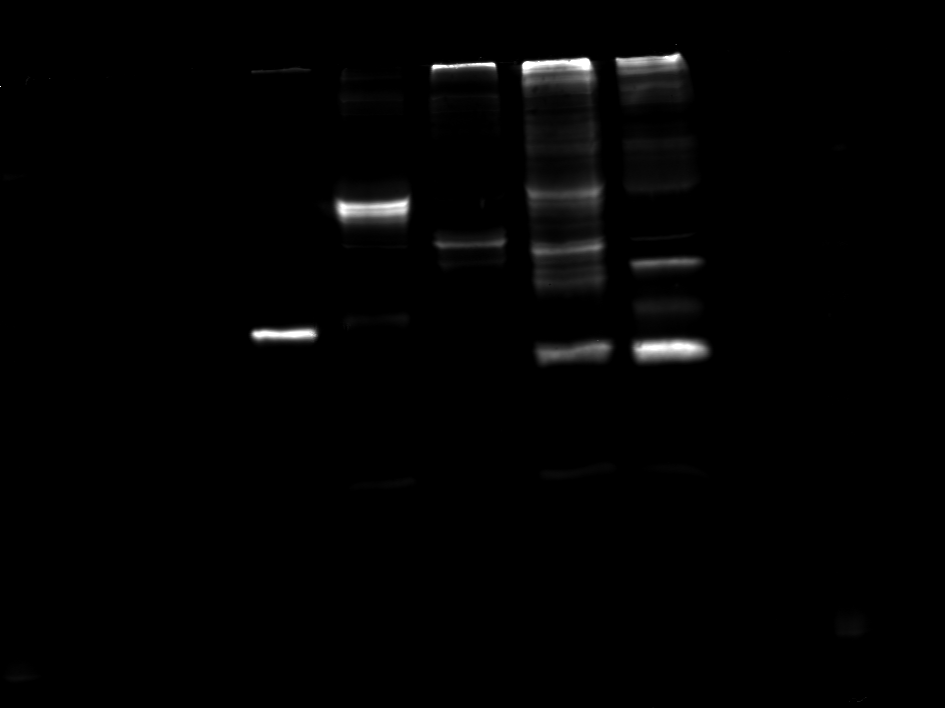


**Figure S14.** *Full-length blot stained for GFP (used for Figure S12).*


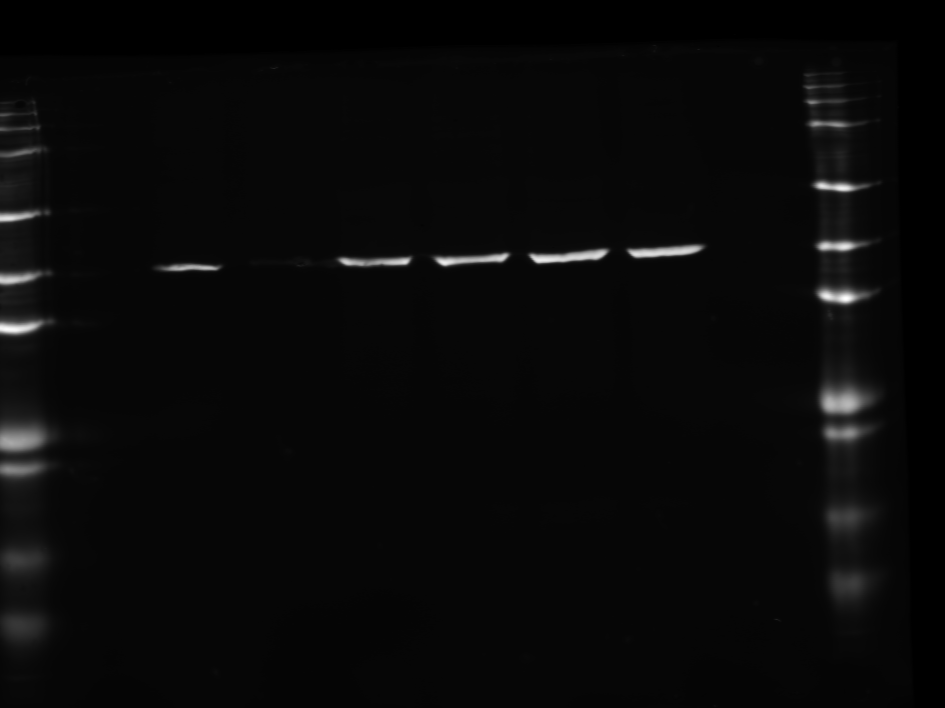


**Figure S15.** *Full-length blot stained for actin (used for Figure S12).*

**References**

1. Mollinedo, F., Martín‐Martín, B., Gajate, C. & Lazo, P. A. Physiological activation of human neutrophils down regulates CD53 cell surface antigen. *J. Leukoc. Biol.* **63**, 699–706 (1998).

2. Angelisová, P., Vlček, Č., Štefanová, I., Lipoldová, M. & Hořejší, V. The human leucocyte surface antigen CD53 is a protein structurally similar to the CD37 and MRC OX-44 antigens. *Immunogenetics* **32**, 281–285 (1990).
